# Body Composition and Body-Image After Spinal Cord Injury: A Scoping Review

**DOI:** 10.3390/nu18213604

**Published:** 2026-10-30

**Authors:** Santina Caliri, Andrea Calderone, Rosaria De Luca, Rosalia Calapai, Alessio Mirabile, Riccardo Raul Ruberto, Angelo Quartarone, Santa Cirmi, Rocco Salvatore Calabrò

**Affiliations:** 1 IRCCS Centro Neurolesi Bonino Pulejo, 98124 Messina, Italy; santina.caliri@irccsme.it (S.C.); rosaria.deluca@irccsme.it (R.D.L.); rosalia.calapai@irccsme.it (R.C.); alessio.mirabile@irccsme.it (A.M.); riccardo.ruberto@irccsme.it (R.R.R.); angelo.quartarone@irccsme.it (A.Q.); roccos.calabro@irccsme.it (R.S.C.); 2 Department of Chemical, Biological, Pharmaceutical and Environmental Sciences, University of Messina, 98100 Messina, Italy; scirmi@unime.it

**Keywords:** spinal cord injury, clinical nutrition, body composition, eating behaviors, body-image, neurorehabilitation, scoping review

## Abstract

**Background/Objectives:** Nutritional changes after spinal cord injury (SCI) can affect bodily appearance and experience, but their relationship with body-image is not consistently defined. This scoping review maps the constructs, measurements, and empirical contributions addressing this intersection. **Methods:** Guidance from the Joanna Briggs Institute and the Preferred Reporting Items for Systematic Reviews and Meta-Analyses extension for Scoping Reviews informed this review. Searches were conducted on 12 September 2026 and were updated on 16 October 2026 across clinical and psychological sources. Two reviewers independently assessed records and reports, with third-reviewer adjudication. Eligible evidence concerned acquired SCI at age 10 years or older or relevant professional perspectives, alongside distinguishable nutritional, and body-image domains. Quantitative associations, co-assessment, and qualitative connections were charted separately with design-specific appraisal and cohort linkage. **Results:** This review includes 36 reports published between 1989 and 2026: 24 contributed patient qualitative findings, 3 contributed professional qualitative perspectives, and 9 contributed quantitative findings. Weight change, perceived muscularity, abdominal appearance, and eating practices were connected with acceptance, attractiveness, and participation. Quantitative evidence combined a small number of direct relational analyses with descriptive co-assessment. Statistical inconsistencies, uncertain measurement properties, and shared samples limited interpretation. Improved satisfaction coexisted with increased adiposity in one uncontrolled report. Adolescent evidence remained sparse. **Conclusions:** Nutritional health and body-image warrant distinct measurement, even when considered together. The evidence identifies experiences and assessment gaps rather than a causal pathway or an effective combined intervention. Longitudinal construct-specific research is needed before the proposed rehabilitation applications can be evaluated.

## 1. Introduction

Spinal cord injury (SCI) affects motor, sensory, and autonomic functions while changing how the body is experienced [1]. Long-term disability and secondary complications make rehabilitation a continuing health priority [2,3]. Nutritional care and body-image intersect within this adjustment, yet a change in body composition does not by itself indicate how a person evaluates their body.

Paralysis-related adiposity and inflammation contribute to neurogenic obesity [4], while altered intake, absorption, and metabolism can create different nutritional risks [5]. SCI-specific cardiometabolic guidance therefore places nutrition within continuing assessment [6]. Concern about excess weight must coexist with attention to undernutrition. Clinical nutrition terminology distinguishes screening, assessment, and diagnosis [7], and the Global Leadership Initiative on Malnutrition requires phenotypic and etiologic criteria [8,9]. Dietary research also identifies discrepancies between intake and SCI-related requirements [10].

Body composition provides information that body weight alone cannot supply. Fat distribution and lean tissue differ across injury characteristics [11], and assessment methods vary in validity and accessibility [12]. Conventional body mass index (BMI) thresholds may underestimate adiposity after SCI [13]. Sarcopenia and sarcopenic obesity require specific clinical criteria [14,15], whose interpretation is complicated by neurological weakness. Perceived thinness, a prominent abdomen, or reduced muscularity cannot independently establish these diagnoses.

Energy expenditure and dietary requirements change after injury [16], with autonomic disruption adding complexity to appetite and metabolic regulation [17]. Dietary guidance consequently requires individual assessment [18]. Eating behavior includes separable dimensions such as emotional eating [19], restraint and disinhibition [20], and attitudes toward bodily eating signals [21]. Similar-looking restrictions can therefore reflect different nutritional, symptomatic, or psychological concerns.

Body-image encompasses perceptions, evaluations, feelings, and behaviors concerning the body. Positive body-image includes acceptance and appreciation [22], while measurement depends on the construct being assessed [23]. Body appreciation [24] and functionality appreciation [25,26] concern dimensions distinct from physical performance. Their application to SCI requires attention to the population and the properties of each score.

Interoception concerns internal bodily sensations and responses to them [27], with accuracy, sensibility, and awareness representing different constructs [28]. These processes may inform hypotheses about eating after SCI, but they do not substitute for evaluating appearance, acceptance, or bodily value. Sensorimotor body schema and ownership likewise require separation from body-image evaluation.

Existing syntheses describe body-image adjustment after SCI through individual, relational, and sociocultural influences [29]. Research across chronic illness examines broader sources of bodily value [30], while disability-wide reviews identify eating-related concerns and developmental assessment needs [31,32]. Their broader populations limit direct inference about SCI, particularly for adolescents and underrepresented groups.

Other reviews address SCI weight-management interventions and stakeholder experiences [33], lifestyle effects on anthropometry and composition [34], and dietary approaches to secondary conditions [35]. What remains less clear is which nutritional and body-image constructs have been examined together, whether their relationship was analyzed directly, and how qualitative accounts connect them. Publications from shared cohorts further complicate the distinction between recurring findings and independent evidence.

The primary objective was to map empirical research jointly assessing, or explicitly connecting, nutritional status, body composition, or eating behaviors and a distinct body-image dimension after acquired SCI. Secondary objectives were to characterize populations, settings, and measurements, distinguish forms of empirical contribution, and identify gaps by age, sex or gender, and study context. Cohort overlap and result-specific limitations were examined to clarify what the evidence can support for nutritional care and neurorehabilitation.

## 2. Materials and Methods

### 2.1. Review Design, Protocol, and Registration

A scoping review was selected to map concepts, measurement approaches, and heterogeneous empirical contributions [36]. Joanna Briggs Institute (JBI) guidance informed the design [37] and protocol specification [38]. Reporting followed the Preferred Reporting Items for Systematic Reviews and Meta-Analyses extension for Scoping Reviews (PRISMA-ScR) [39].

The review was conceived as a scoping review from the outset. The protocol was deposited on the Open Science Framework (OSF) on 11 September 2026 under project identifier XCBHR and DOI 10.17605/OSF.IO/XCBHR. The initial search was conducted on 12 September 2026 and the final search update on 16 October 2026. The chronology distinguishes the protocol deposit, initial search, and updated retrieval rather than a change of review design. Supplementary Table S1 summarizes the design, chronology, and responsibilities.

Santina Caliri (S.Ca.) and Andrea Calderone (A.C.) were the independent reviewers for searches and exports, screening, full-text assessment, charting, appraisal, and synthesis checks. Assessments were compared after independent consideration. Discussion resolved disagreements where possible, and Rocco Salvatore Calabrò (R.S.C.) adjudicated unresolved differences. These responsibilities also applied to the updated search and extraction.

### 2.2. Review Question and Eligibility Criteria

The review question was: What empirical research jointly assesses, or explicitly connects, nutritional status, body composition, or eating behaviors and a distinct body-image dimension in people aged at least 10 years with acquired SCI? Secondary questions concerned populations, contexts, measurements, and evidence gaps. Eligibility followed the Population, Concept and Context (PCC) framework [40].

The population comprised adolescents and adults with acquired traumatic or non-traumatic SCI, including separately identifiable eligible subgroups of mixed samples. Professional or caregiver perspectives were considered separately when their findings explicitly addressed the same population and conceptual intersection.

The concept required an identifiable nutritional domain and a distinct perceptual, evaluative, or experiential body-image dimension. Eligible forms of contribution were quantitative association analysis, quantitative co-assessment, and explicit qualitative connection.

The context included acute care, rehabilitation, and community living in any country and at any time since injury. No particular intervention or comparator was required.

Eligibility concerned each report and its extractable contribution. Co-assessment meant that distinguishable constructs were measured in the same eligible sample, whereas an association required a relational analysis. Qualitative evidence required an explicit connection in the findings. Supplementary Table S2 specifies these boundaries.

#### 2.2.1. Inclusion Criteria

Eligible patient findings concerned acquired SCI at age 10 years or older at assessment, irrespective of injury level, completeness, duration, mobility, sex, or gender. Mixed samples required separately attributable findings meeting both age and diagnostic criteria. Professional and caregiver accounts were eligible only for explicit empirical perspectives on the same intersection.

The nutritional domain included status or risk, intake, measured or self-reported weight, recalled weight change, anthropometry, composition, and eating practices or attitudes governing intake. Perceived fatness, thinness, or muscularity were retained as subjective descriptors. A separate body-image dimension could concern perceived size, appearance evaluation, acceptance, appreciation, body esteem, self-presentation, or satisfaction with functionality.

Quantitative contributions required separate assessments, instruments, items, or subdomains within the same identifiable eligible sample. Perceived body size could qualify alongside anthropometry without implying dissatisfaction. Association tests, comparators, and statistical significance were not inclusion requirements.

Qualitative findings required an explicit connection between distinguishable domains within participant accounts, themes, or empirical results. Recalled weight change or perceived muscle loss could therefore qualify alongside a distinct bodily evaluation, without being converted into measured composition or a nutritional diagnosis.

Each independently eligible report required its own extractable contribution. Companion publications could supply context when the cohort, subgroup, and assessment period corresponded. Combining unrelated nutritional and body-image publications did not establish co-assessment. Shared participants were linked without counting them as additional independent observations.

Eligible sources were original full-length journal articles in English, including official English versions. Observational, intervention, measurement-property, qualitative, and mixed-methods research could qualify. Publication labels alone did not determine design: a multi-participant analytical report was distinguished from a clinical case report. No lower publication-date boundary, minimum sample size, or quality-score threshold was imposed.

#### 2.2.2. Exclusion Criteria

Participants younger than 10 years, congenital conditions, and isolated peripheral nerve or nerve-root disorders were excluded unless eligible acquired-SCI findings were separately identifiable. An adult mean age or a majority SCI diagnosis did not resolve an inseparable mixed sample.

Reports lacking either domain were excluded. Physical activity, strength, feeding dependence, food access, or nutrition knowledge alone did not establish a nutritional contribution. General quality of life, mood, global self-esteem, sexual function, motor performance, body schema, or ownership alone did not establish body-image. A single weight-dissatisfaction item could not serve as both an independent nutritional indicator and a body-image outcome.

Qualitative connections confined to background or discussion sections were insufficient. Measures from unrelated samples did not constitute co-assessment. Shared authors, institutions, or sample size did not alone establish participant overlap.

Reviews, protocols, editorials, conference abstracts, dissertations, standalone preprints, clinical case reports, and reports without an English full text were excluded. Qualitative studies were classified by their research design rather than participant numbers. Non-retrieval and unresolved eligibility were distinguished from substantive exclusion.

### 2.3. Information Sources and Search Strategy

Information sources comprised PubMed, Web of Science Core Collection, Embase, the Cochrane Library, Scopus, APA PsycINFO, and CINAHL (accessed through EBSCOhost). Cochrane exports included CENTRAL and other Library collections; a separate Embase export supplied trial-registration records. Registry platforms were not searched directly. PRISMA-S informed search reporting [41]. Supplementary Table S3 reproduces the source-specific expressions, yields, and documentation.

The intended textual structure was SCI AND (body-image OR (nutrition AND psychosocial/experiential terms)). The body-image branch did not require nutritional terminology, allowing for potentially relevant information to appear only in the full text. The second branch sought nutritional studies with broader psychological or experiential language. Retrieval terms such as quality of life or self-esteem did not replace the distinct body-image eligibility requirement.

The expressions retain the syntax and field labels recorded by each query builder. Web of Science used Topic (TS), and APA PsycINFO used Any Field; the supplied PubMed and Scopus expressions were untagged. CINAHL was searched through the EBSCOhost platform. Searches had no lower publication-date boundary. English-language eligibility concerned the full report rather than the language of an abstract or participant interview.

Searches covered database inception to the final update of 16 October 2026. S.Ca. and A.C. checked strategies and exports independently, with R.S.C. adjudicating unresolved differences. PRESS guidance informed checks of concepts, Boolean structure, terms, fields, and limits [42]; this was not presented as a separately documented formal PRESS review. Known-report coverage and reference checks were considered alongside database retrieval. Search volume was not treated as proof of exhaustive or equivalent coverage.

### 2.4. Record Management and Study Selection

Deduplication prioritized persistent identifiers, followed by title, authors, year, and journal, with uncertain matches inspected using published principles [43]. Overlap between the two Scopus export segments was reconciled before bibliographic deduplication. Separate publications from shared cohorts retained their identities.

Rayyan supported record management, manual decisions, relevance ordering, and automated exclusions. Every title/abstract record, including records affected by automated functions, was subject to the independent assessment reported by S.Ca. and A.C. Rayyan-assisted exclusions were counted within title/abstract screening rather than added again as removals before screening. Statistical significance did not determine eligibility.

S.Ca. and A.C. independently assessed all titles/abstracts and all full-text reports before comparison, rather than reviewing only ambiguous records. Discussion and, when needed, R.S.C. adjudication resolved disagreements. One primary reason was assigned per excluded record or report. Full-text reasons considered publication type, language, population, nutritional domain, body-image domain, and empirical contribution. Supplementary Table S4 separates the decision rules, stage-specific categories, and reconciled counts.

### 2.5. Data Charting and Source Verification

Charting followed guidance on extracting and presenting scoping-review evidence [44]. Fields covered publication identity, setting, design, recruitment, age, sex or gender, injury characteristics, analytical sample, respondent perspective, nutritional and body-image constructs, instruments, timing, and findings. Supplementary Table S5 describes the procedures. Missing information was retained as not reported.

S.Ca. and A.C. independently charted and checked substantive entries against the primary publications before reconciliation, with R.S.C. adjudicating unresolved differences. Journal-issue years were used when assigned. Enrollment, completers, eligible subgroups and result-specific observations were distinguished. Supplementary Table S6 records the detailed findings and denominators supporting the main tables.

Consensus-based Standards for the Selection of Health Measurement Instruments (COSMIN) guidance informed interpretation of measurement properties and subscales [45]. Total questionnaire reliability was not attributed to a body-image subdomain. Separate items or scores remained distinct even when collected within one instrument.

Cohort linkage required explicit recruitment descriptions or matching participant and assessment information. Parent-cohort measurements were not assigned to interviewed subgroups without documented correspondence. Uncertain overlap remained uncertain rather than being interpreted as independence.

Coefficients, probability values, timing, and analytical denominators were checked together. Unclear denominators were not reconstructed from enrollment or repeated visits. Conflicting published statistics were retained as reporting uncertainties rather than silently repaired. Qualitative findings preserved eligible participant and contextual attribution; background or discussion statements were not extracted as empirical results.

### 2.6. Critical Appraisal

Appraisal was matched to the empirical contribution using the declared JBI qualitative (2017), analytical cross-sectional (2017), cohort (2020), and case-series (2020) checklists [40,46]. The revised quasi-experimental tool (2024) addressed uncontrolled intervention change [47]. COSMIN judgments concerned the measurement properties actually investigated. Astorino and Harness received separate appraisals for pre-/post-change and longitudinal associations within one report.

Qualitative appraisal addressed methodological congruity, researcher positioning, participant representation, ethics, and support for conclusions. Quantitative appraisal considered selection, measurement, confounding, follow-up, missing data, and analysis. Unclear reporting was distinguished from a demonstrated flaw, and measurement-property judgments did not become a study-wide quality category.

Supplementary Table S7 presents the item-level and property-specific judgments with their interpretive consequences. JBI categories were Yes, No, Unclear, and Not applicable; COSMIN ratings remained property-specific. S.Ca. and A.C. were responsible for independent appraisal and reconciliation, with R.S.C. adjudication where required. No summed score, numerical exclusion threshold, or quality weighting was used. Confounding and temporal ambiguity constrained relational claims, measurement limitations constrained construct interpretation, and qualitative reporting informed assessment of context and transferability.

### 2.7. Data Synthesis and Evidence Mapping

Synthesis combined descriptive mapping and qualitative content organization [48]. Categories covered nutritional status and intake, weight and composition, perceived bodily change, eating practices, appearance, acceptance, and functionality. Patient and professional contributions were organized separately. The review did not reanalyze original interview transcripts.

Quantitative findings were classified as direct relational analyses, co-assessment, or parallel changes. Units, denominators, timing, and uncertainty were retained. Qualitative connections described meanings grounded in the reported findings rather than prevalence or causal pathways.

The report was the bibliographic unit. Linked cohorts, nested subgroups, and repeated assessments were tracked separately, as summarized in Supplementary Table S8. No meta-analysis, significance-based vote counting or pooled certainty assessment was undertaken. The heterogeneous constructs and sparse direct relational evidence did not support one common effect estimate.

Contrasting accounts and absent measurements were retained without imputation. Supplementary Table S9 links measurement and reporting limitations with their implications for synthesis. Evidence gaps concerned population coverage, construct assessment, and the questions actually investigated.

### 2.8. Reporting Transparency and Clinical Interpretation

PRISMA terminology distinguished records, reports, and the investigations they described [49]. Non-retrieval was separated from substantive ineligibility, and report counts were not equated with independent cohorts. Numerical reconciliation did not substitute for record-level eligibility assessment.

Interpretation followed guidance on challenges in scoping reviews [50]. Contextual literature was distinguished from the included empirical corpus. Hypotheses about psychological or neuropsychological processes were not treated as findings unless those processes had been assessed.

The rehabilitation framework and assessment scenarios were conceptual applications developed by the authors. Their feasibility, acceptability, and effects require evaluation; they were not interventions validated by the mapped studies.

## 3. Results

### 3.1. Study Selection

The retrieval identified 26,897 records: 26,615 bibliographic-database records plus 282 trial-registration records from a separate Embase export. The yields were as follows: PubMed, 514; Web of Science, 470; Embase journal records, 1109; Scopus, 23,993; APA PsycINFO, 15; CINAHL (EBSCOhost), 311; and the Cochrane Library, 203. Screening covered 24,642 records of which 24,053 were excluded with one primary reason per record.

The database branch sought and assessed 589 reports, with no reports remaining unretrieved at completion of eligibility assessment. Its 555 exclusions comprised publication type (21), language (17), population (71), absent nutritional domain (109), absent distinct body-image domain (118), absent required empirical connection (12), and outside review scope (207). Thirty-four reports were retained from this branch.

**Figure 1:**
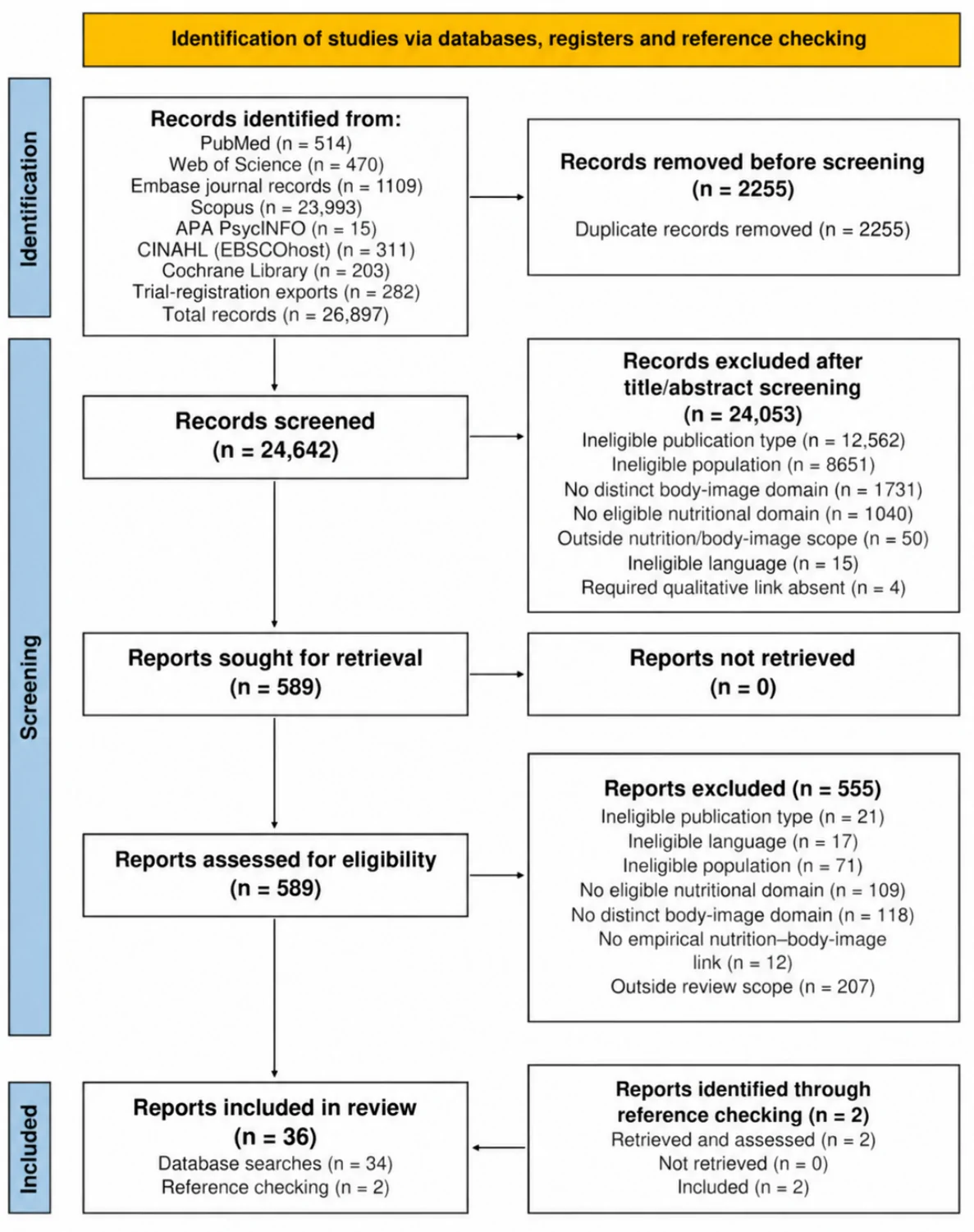
PRISMA flow diagram for the updated searches and study selection. The 26,897 source records yielded 24,642 records after bibliographic deduplication. Title/abstract screening excluded 24,053 records, leaving 589 reports for eligibility assessment. Database screening yielded 34 included reports, with 2 additional reports identified through reference checking, for 36 eligible reports. CINAHL was accessed through EBSCOhost. Exclusions are assigned one primary reason. Reports and independent cohorts remain distinct units.

Reference checking identified two further eligible reports from the original corpus that were absent from the exports. The combined set comprised 36 included publications after 591 report assessments across the two branches. Figure 1 summarizes the identification, screening, and selection of eligible reports. These are publication counts, not independent-cohort counts.

### 3.2. Characteristics of the Included Reports and Cohorts

Thirty-six reports were published between 1989 and 2026. Twenty-four contributed patient qualitative findings, three contributed professional qualitative perspectives, and nine contributed quantitative findings, classified by the principal eligible contribution rather than every component of a parent investigation. Table 1 summarizes their characteristics.

North America contributed twenty-one reports (58.3%), Europe contributed seven (19.4%), Oceania contributed four (11.1%), Asia contributed two (5.6%), Africa contributed one (2.8%), and South America contributed one (2.8%). Twelve national settings were identifiable; Perrier et al. described a North American sample without itemizing participants’ countries [82]. These distributions use publications as their denominator and do not indicate independent national replication.

The qualitative contributions comprised 24 patient reports (66.7%) and 3 provider reports (8.3%). Desneves et al. contributed patient interviews within a mixed-methods implementation study [69]. The nine quantitative reports (25.0%) included longitudinal, cross-sectional, retrospective and measurement-property investigations. Direct nutritional–body-image analyses were a subset of this quantitative evidence rather than a requirement met by all nine reports.

Seven reports concerned women-only patient samples or eligible subgroups and four concerned men-only samples. Two specifically examined adolescents or adolescents and young adults [74,75]. These report-level counts do not describe a sex-balanced participant pool. Eligibility-specific subgroup information was retained when mixed-sample ages or sex distributions could not be transferred to SCI participants.

Recruitment included community organizations, veterans’ systems, specialist SCI centers, and acute or rehabilitation wards. Provider-only interviews added perspectives on weight management and diet, while patient accounts described lived experiences. Neither respondent group was treated as a substitute for the other.

Several reports described shared participants. Bailey’s positive-body-image and self-presentation analyses used the same nine-person sample [58,59]. Sheldon’s male sample and Renwick’s analysis arose from a shared parent project [63,64]. LaVela’s healthy eating and weight-goal reports involved closely corresponding interviews with 25 adults [54,79]. Possible provider-sample reuse was retained as uncertain where linkage was incomplete. No unique participant total could be reconstructed reliably.

**Figure 2:**
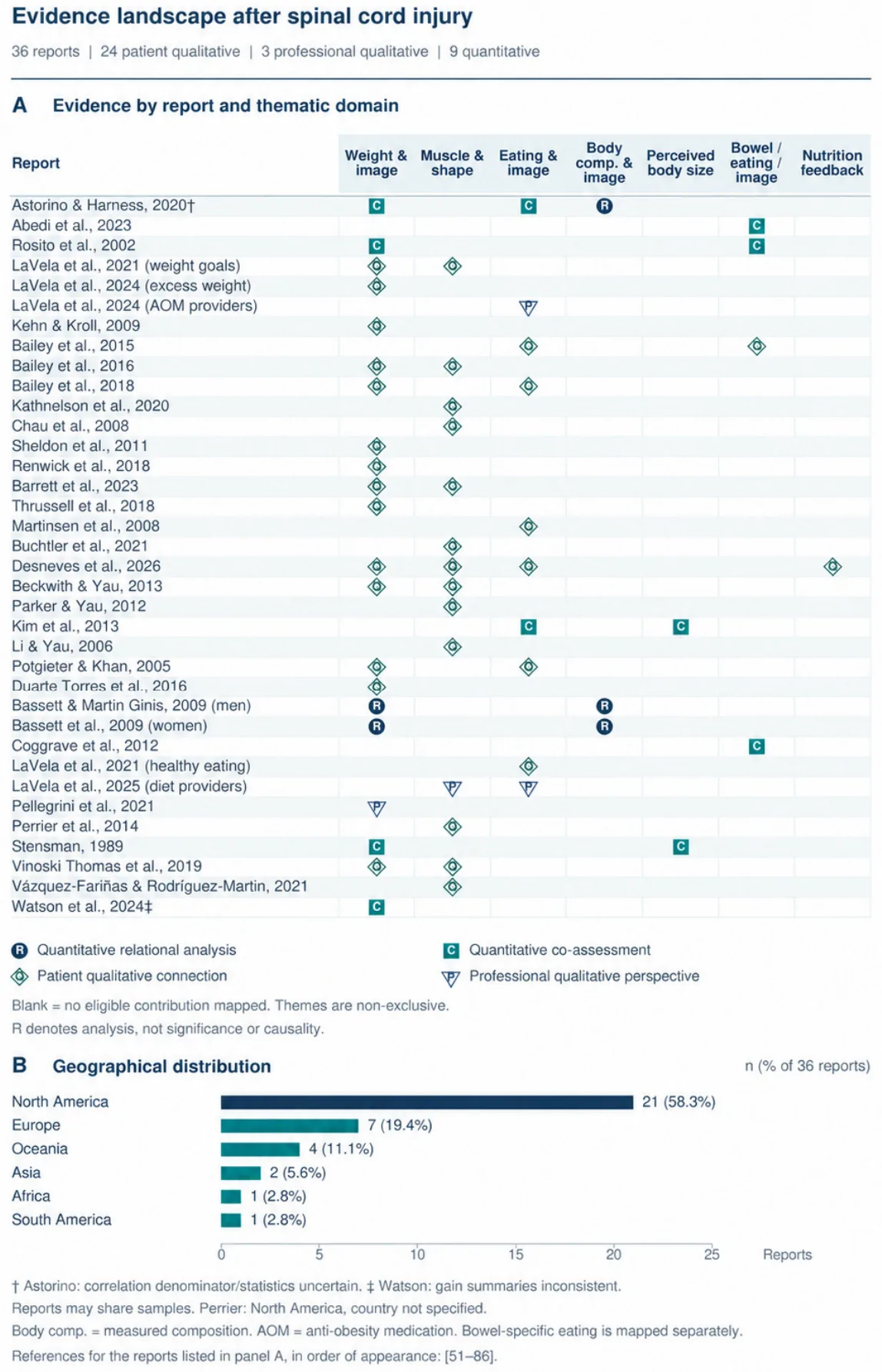
Evidence landscape across 36 eligible reports. (**A**) The matrix distinguishes quantitative relational analyses (R), quantitative co-assessment (C), patient qualitative connections (Q), and professional perspectives (P). Blank cells indicate no mapped contribution to that domain. Themes are non-exclusive. Relational analysis does not imply a significant association or causation. Symbols † and ‡ retain the statistical and reporting uncertainties for Astorino and Watson. (**B**) Geographical percentages use 36 reports. Perrier remains classified as North America without a specified country. Shared or potentially overlapping samples are documented in Supplementary Table S8. Reports in panel A are listed in the order of references [51,52,53,54,55,56,57,58,59,60,61,62,63,64,65,66,67,68,69,70,71,72,73,74,75,76,77,78,79,80,81,82,83,84,85,86].

The separation of report identity, cohort linkage, and eligible empirical contribution informed the evidence map summarized in Figure 2.

### 3.3. Nutritional Status, Body Composition, and Body Perception

Measured composition and bodily evaluation did not necessarily change together. Astorino and Harness reported six-month follow-up in 25 of 29 recruited participants [51]. Mean Body Satisfaction Survey (BSS) increased from −0.36 to 0.44, while body fat increased from 32.7% to 33.8% and reported energy intake decreased from 1769 to 1661 kcal/day. Their published body-fat/ΔBSS result, r = 0.33 and *p* = 0.049, has an unclear analytical denominator and does not match a conventional two-sided correlation test using 25 independent observations. The related r = 0.36, *p* = 0.03 result is also uncertain. Values were retained as reported, without assuming pooled visits or correcting the source. Table 2 summarizes constructs, findings, and contribution types.

The two SHAPE-SCI reports provided separate anthropometric and satisfaction assessments. The women-only pilot (n = 11) reported an inverse appearance-satisfaction/waist correlation, Spearman ρ = −0.64, *p* < 0.05, using one-tailed exploratory tests [77]. Its body-fat correlation, ρ = −0.46, met only *p* < 0.10. The men-only report (n = 50; waist n = 49) reported nonsignificant nutritional–body-image correlations [76]. Small samples, multiple comparisons, and different analyses precluded a reliable sex comparison or pooled relationship.

Other quantitative reports primarily mapped co-assessment. Kim et al. enrolled 371 participants; 42 of the 84 classified with abdominal obesity perceived themselves as fat or very fat [72]. Internal denominator inconsistencies prevented use of the reported prevalence as 84/371. Stensman separately identified ten participants with traumatic tetraplegia within a mixed sample, with BMI and body-image ratings but no specific BMI–image association test [83]. Watson et al. assessed separate perceived weight-management and appearance gains [86]. These composite reports did not measure weight loss, and inconsistent pooled/subgroup summaries limited numerical interpretation.

Bowel-related studies provided further co-assessment. Abedi et al. retained distinct food-avoidance and body-image items in analyses involving 106 patients [52]. Overall IBS-QOL internal consistency and correlations with bowel-related comparators did not establish domain-specific validity or a nutritional–body-image association. Rosito et al. reported recalled weight loss in 8/27 respondents before stoma and 2/27 afterward, with body-image change described as nonsignificant despite an irregular printed probability [53]. Coggrave et al. analyzed 92 ostomates, assessing dietary manipulation separately from appearance and physical self-concept [78]. Neither stoma report tested whether eating or weight changes explained body-image outcomes.

Desneves et al. illustrate the distinction between clinical and interview samples [69]. Body-composition measurements concerned 21 patients, whereas 12 were interviewed and five clinicians joined a separate focus group. Interview accounts connected weight or muscle changes and dietary feedback with bodily evaluation. Aggregate clinical values could contextualize those accounts, but could not be assigned to individual interviewees or treated as corresponding associations.

### 3.4. Eating Behaviors and Body-Image Experiences

Eating practices entered both self-care and appearance-related efforts. Bailey et al. described eating and bodily routines within positive body-image [58]. Their separate dietary-intervention follow-up involved six unique participants, with some contributing to both individual interviews and a focus group [60]. Recalled weight loss and appearance benefits were experiential findings rather than measured effects in that subgroup. Martinsen et al. connected food choices, assistance, and meal devices with dignity and self-presentation among 16 people with high cervical SCI [67].

LaVela’s weight-management interviews connected appearance, clothing fit, health, and mobility with desired weight goals [54], while excess-weight accounts described emotional and physical burden [55]. The healthy eating report added a narrow skin-appearance belief rather than a global body-image assessment [79]. A companion nutrition publication was retained as contextual literature because its eligible, distinct, body-image contribution was not established [87]; it did not add an independent inclusion or population.

Provider reports described a different level of evidence. Pellegrini et al. interviewed 25 professionals and identified body-image concerns within psychological barriers to weight management [81]. LaVela reports that 12 providers each linked altered body habitus, eating concerns, and perceived vulnerability during obesity pharmacotherapy [56,80]. These were professional interpretations, not measured patient outcomes, eating-disorder prevalence, or medication effects. Potential overlap between the provider samples further limited independent confirmation.

### 3.5. Psychosocial Context and Rehabilitation-Related Findings

Appearance concerns involved perceived smallness, as well as excess weight. Potgieter and Khan’s seven adolescent and young-adult participants connected remembered weight loss and thinness with attractiveness and sexual self-esteem [74]. Duarte et al. similarly described bodily change in a small adolescent/young-adult sample [75]. Age-range inconsistencies in the latter report prevented more precise developmental attribution.

Muscularity and abdominal appearance were recurrent experiential concerns. Bailey et al. described gendered expectations and impression management [59], while Kehn and Kroll situated weight loss and regain within exercise narratives [57]. Kathnelson et al., Buchtler et al., and Barrett et al. described changes in attractiveness, self-recognition, or confidence [61,65,68]. The meaning and direction of evaluation varied across people and situations.

The qualitative accounts also connected bodily change with recognition and self-presentation. Vázquez-Fariñas and Rodríguez-Martin linked perceived leg thinning with negative appearance evaluation and avoidance of shorts [85]. Vinoski Thomas et al. provided separately identifiable accounts from five women with SCI within 15 women with mixed disabilities [84]. Perrier et al. included nine participants with SCI within eleven participants and an explicit visual and emotional response to perceived leg atrophy [82]. Findings from other disability groups were not transferred to SCI.

Clothing, mirrors, and intimacy made bodily changes socially consequential. Chau et al. described abdominal appearance and clothing choices [62], Beckwith and Yau reported changed muscular contour and attractiveness [70], and Parker and Yau examined abdominal shape and sexual identity [71]. Li and Yau’s eligible SCI accounts were separated from poliomyelitis accounts [73]. These descriptions did not establish measured tissue loss or sarcopenia.

Sheldon’s 64-man analysis linked perceived thinness with clothing and self-presentation, while Renwick’s 30-man analysis drew on the same parent project [63,64]. Thrussell et al. included an identifiable thoracic-SCI account of weight gain and mirror or photograph avoidance within a mixed SCI/CES sample [66]. Its inconsistent subgroup descriptions prevented reconstruction of one exact eligible sample denominator.

The qualitative findings therefore documented meanings, settings, and individual priorities. They did not establish the physical magnitude of change, its frequency in the wider SCI population or whether nutritional change preceded altered body evaluation.

### 3.6. Methodological Appraisal and Evidence Gaps

**Figure 3:**
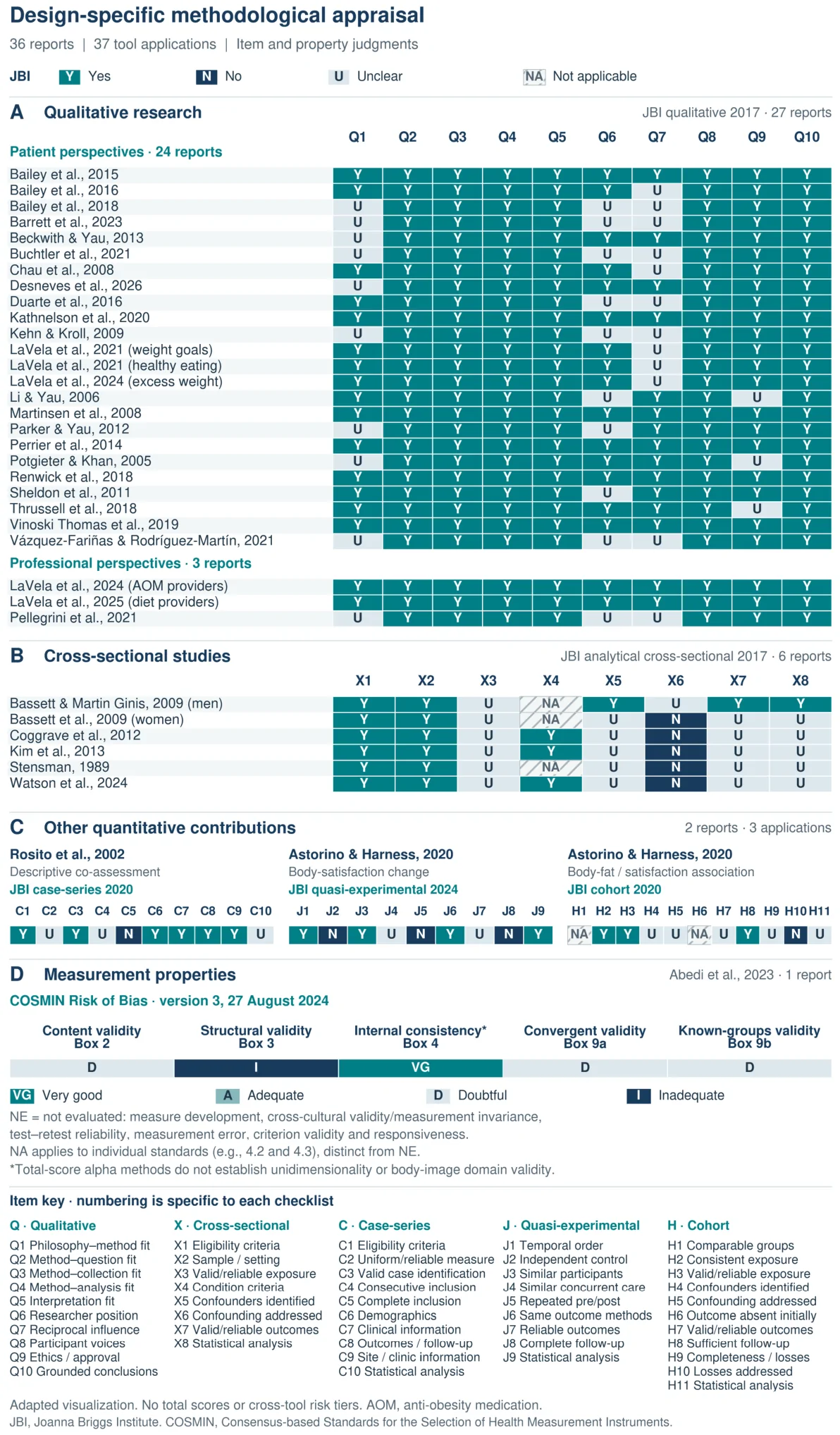
Design-specific methodological appraisal of 36 reports using 37 tool applications. (**A**) Qualitative, (**B**) cross-sectional, (**C**) other quantitative, and (**D**) measurement-property appraisals are presented separately. Astorino and Harness contribute two distinct appraisals. Tool versions, categories, and item definitions are shown. Ratings do not constitute a common score. The rating for calculating Abedi’s total alpha does not establish body-image domain validity. The reports shown are Astorino and Harness, 2020 [51]; Abedi et al., 2023 [52]; Rosito et al., 2002 [53]; LaVela et al., 2021 (weight goals) [54]; LaVela et al., 2024 (excess weight) [55]; LaVela et al., 2024 (AOM providers) [56]; Kehn and Kroll, 2009 [57]; Bailey et al., 2015 [58]; Bailey et al., 2016 [59]; Bailey et al., 2018 [60]; Kathnelson et al., 2020 [61]; Chau et al., 2008 [62]; Sheldon et al., 2011 [63]; Renwick et al., 2018 [64]; Barrett et al., 2023 [65]; Thrussell et al., 2018 [66]; Martinsen et al., 2008 [67]; Buchtler et al., 2021 [68]; Desneves et al., 2026 [69]; Beckwith and Yau, 2013 [70]; Parker and Yau, 2012 [71]; Kim et al., 2013 [72]; Li and Yau, 2006 [73]; Potgieter and Khan, 2005 [74]; Duarte et al., 2016 [75]; Bassett and Martin Ginis, 2009 (men) [76]; Bassett et al., 2009 (women) [77]; Coggrave et al., 2012 [78]; LaVela et al., 2021 (healthy eating) [79]; LaVela et al., 2025 (diet providers) [80]; Pellegrini et al., 2021 [81]; Perrier et al., 2014 [82]; Stensman, 1989 [83]; Vinoski Thomas et al., 2019 [84]; Vázquez-Fariñas and Rodríguez-Martín, 2021 [85]; and Watson et al., 2024 [86].

Appraisal identified result-specific limits rather than one common quality level. Ten of twenty-seven qualitative contributions had unclear congruity between philosophical perspective and methodology, ten unclear researcher positioning, and twelve unclear reporting of researcher influence. These reporting gaps constrained contextual interpretation without establishing that participant accounts were invalid. Quantitative concerns included confounding, small samples, recall, missing denominators and uncertain measurement properties. Figure 3 presents the item-level and property-specific appraisals.

Appraisal affected the interpretation of particular findings. Uncontrolled Astorino changes could not establish therapy effects, exploratory Bassett correlations did not support stable population estimates, and the Abedi total alpha did not validate a body-image subscale [51,52,76,77]. Inconsistent Watson summaries limited magnitude estimates rather than being silently repaired [86]. Table 3 integrates the resulting themes, evidential limits, and research questions.

Few investigations measured both domains distinctly, analyzed their relationship directly, and established temporal ordering. Adolescent evidence, representative gender comparisons, and repeated assessment across rehabilitation stages were limited. No sufficiently comparable independent estimates supported pooling. The map therefore describes experiences and measurement opportunities more securely than mechanisms or effective interventions.

## 4. Discussion

### 4.1. Nutritional Change and Bodily Meaning

The map complements reviews of dietary adequacy [10], composition assessment [12], body-image adjustment [29], and weight-management interventions [33,34] by distinguishing the form and population of each contribution. Its central finding is that nutritional descriptors and bodily evaluations can coexist, diverge, or become explicitly connected in personal accounts. The predominance of qualitative evidence is informative about experience, but leaves the magnitude, temporal direction, and independent replication of most relationships unresolved.

The contrast between measured composition and satisfaction is particularly instructive. Astorino’s parallel changes do not show that adiposity improves satisfaction [51]. The exploratory SHAPE-SCI correlations likewise cannot establish that altering waist circumference changes appearance evaluation [76,77]. A person can report greater comfort or acceptance while clinical risk persists, or improved clinical measurements while remaining distressed. Group means cannot establish these individual trajectories, which require linked repeated observations.

Nutritional care consequently needs its own clinical rationale. SCI nutrition syntheses address metabolic and gastrointestinal consequences [88], and a position paper and expert consensus emphasize assessment across care settings [89,90]. Longitudinal malnutrition research [91] and findings in older people with traumatic cervical SCI [92] show why desired thinness cannot determine an appropriate nutritional goal. The direction of care may involve adequacy, restoration, or management of excess adiposity according to the person’s clinical situation.

Longitudinal composition findings and energy-expenditure research further demonstrate variation hidden by weight alone [93,94]. Bowel symptoms and pressure-injury care can introduce priorities that differ from appearance goals [95,96]. A dietitian-led randomized trial found no clear between-group difference in its primary total-fat-mass endpoint, although secondary visceral-fat and waist outcomes favored intervention [97]. None of these contextual findings demonstrates improved body-image acceptance. Clinical and experiential outcomes should therefore remain separate endpoints.

### 4.2. A Conceptual Basis for Coordinated Assessment

The mapped accounts suggest questions for coordinated assessment rather than a validated rehabilitation pathway. A concern about abdominal size might prompt clarification of weight history, intake, bowel symptoms, posture, and assistance, alongside questions about clothing, intimacy, and participation. The aim would be to identify which difficulty matters to the person and which measurements can address it. Nutritional findings would not be used as proxies for distress, nor would appearance concerns alone determine a weight target.

The feasibility of any response depends on everyday circumstances. Meal-preparation and shopping studies identify practical reliance on assistance [98,99], while food-access research shows that structural disadvantage cannot be reduced to BMI [100]. The reasons for ending meals also differ after SCI [101]. A skipped meal could reflect symptoms, scheduling, appetite, support, or appearance-related avoidance. Each explanation suggests a different question for assessment rather than one assumed psychological mechanism.

Intervention evidence must retain its own population and outcomes. Telenutrition protocol development informs content and delivery, not completed effectiveness [102]. The metabolic and microbiome reports by Li et al. arose from the same trial [103,104]; they do not provide independent evidence of psychological benefit. Preliminary glucagon-like peptide-1 (GLP-1) receptor agonist observations concern a very small controlled case series [105]. Nutrition education [106] and established bowel guidance [107] can inform clinical care without validating the combined framework proposed here.

Supplementary Table S10 translates the evidence into illustrative assessment scenarios. Possible evaluation outcomes include nutritional adequacy, body-related distress, acceptability, and personally valued participation, assessed separately. Feasibility, implementation demands, and adverse consequences would need examination before any standardized integrated pathway could be recommended.

### 4.3. Psychological Explanations That Remain to Be Tested

SCI research identifies cognitive and emotional difficulties that can influence adjustment [108,109]. These findings justify considering attention, memory, mood, and the social context of eating, but they do not explain every appearance concern. Difficulties following a dietary plan require assessment before being attributed to impaired cognition; perceived cognitive functioning and performance can show different symptom associations [110].

Interoceptive and embodied-cognition accounts offer testable explanations for some experiences [111,112]. Their relevance to the present map remains hypothetical because most included reports did not measure the proposed processes. Eating-disorder neuroimaging in other populations cannot establish the same mechanism after SCI [113]. A useful study would need a defined nutritional exposure, a distinct body-image outcome and a measured candidate mechanism, with temporal ordering and plausible competing explanations.

Intervention targets require similar restraint. Associations between acceptance-based processes and distress after SCI are cross-sectional [114], while reviews of mindfulness and acceptance research identify heterogeneous methods [115]. Mental-health guidance supports assessment and treatment of recognized disorders [116], but the present review does not establish an eating/body-image treatment. Adaptations would require explicit targets, appropriate comparators, and outcomes beyond general well-being.

**Figure 4:**
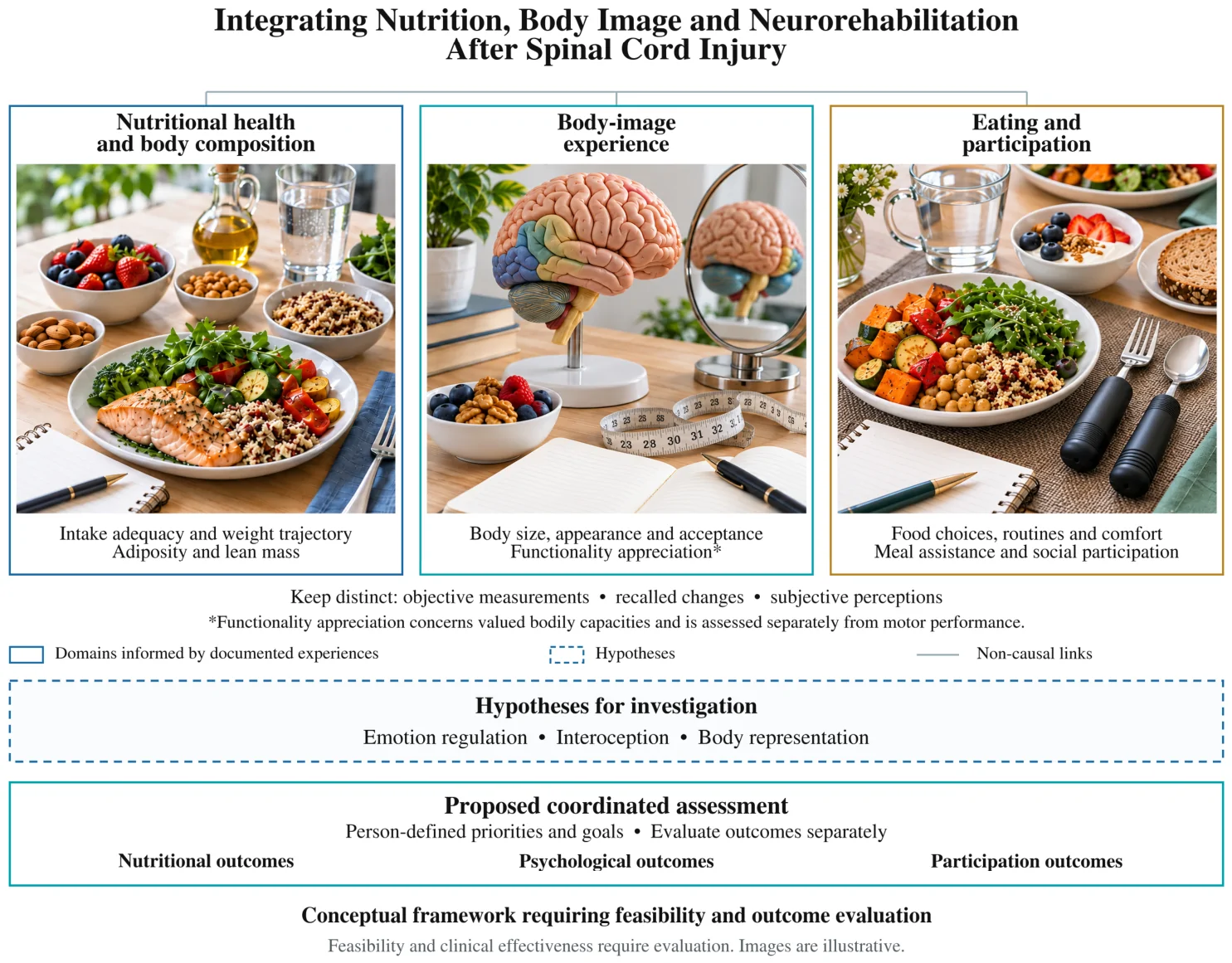
Conceptual framework for possible coordinated assessment after spinal cord injury. Solid-framed domains concern nutritional health and body composition, body-image experience, and eating and participation. Undirected lines indicate domains for joint consideration. The dashed area contains hypotheses for investigation. Objective measures, recalled changes, and subjective perceptions remain distinct, as do functionality appreciation and motor performance. Person-defined priorities guide evaluation of separate nutritional, psychological, and participation outcomes. Feasibility and clinical effectiveness require evaluation. The scenes are illustrative, and the brain model does not represent a measured neural mechanism.

Functionality appreciation and self-compassion may help formulate research questions, although the broader literature differs in populations, design, and follow-up [117,118]. A small SCI peer-mentor program provides feasibility information rather than evidence of nutritional or body-image efficacy [119]. Figure 4 separates the three assessment domains from psychological processes proposed for investigation. The framework is a conceptual application of the evidence map, not a tested causal model.

### 4.4. Limits of the Map and Priorities for Research

This review distinguishes quantitative analyses of relationships between nutritional and body-image variables from studies that measured both domains without testing their relationship and qualitative accounts describing connections between them. This distinction clarifies what each type of evidence contributes without treating co-assessment as evidence of association. Reports arising from shared or potentially overlapping cohorts provide complementary perspectives, but cannot be regarded as independent confirmation.

Search sensitivity may have been constrained by differences in terminology and database indexing, together with the contextual terms required in the nutritional search branch. Restrictions to English-language journal publications may have excluded relevant evidence and experiences reported in other languages or publication formats. These constraints limit the completeness of the evidence map and should be considered when interpreting its scope and applicability.

The 27 qualitative contributions provide detailed accounts, but cannot establish prevalence or stable effect sizes. Specialist-service recruitment, veterans’ samples, and North American concentration constrain transferability. Only two reports specifically addressed adolescents or adolescents and young adults [74,75]. Sex-specific samples were uneven, and mixed-disability reports sometimes lacked subgroup demographics. Developmental stage, gendered expectations, and cultural setting therefore remain important gaps rather than established moderators.

Measurement and reporting limitations also shaped the conclusions. Subjective morphology, recalled weight, and objective composition are different indicators. Body-size perception, appearance satisfaction, acceptance, and questionnaire body-image items are not interchangeable outcomes. Uncertain denominators, statistical inconsistencies, and unlinked clinical/interview samples constrain relational interpretation, even when the source publication is correctly identified.

Future studies should link repeated nutritional and construct-specific body-image assessments within the same participants, report missingness and analysis-specific denominators, and identify reused cohorts. Adequately designed observational studies can examine confounding and temporal direction. Mixed-methods work can connect individual trajectories with personal meanings, while adolescent and culturally diverse sampling can test the reach of findings currently grounded mainly in adult accounts.

Intervention development should involve people with SCI in selecting acceptable goals and outcomes. Separate assessment of nutritional adequacy, bodily experience, and participation would allow benefit in one domain to be recognized without assuming improvement in another. Digital delivery may support access, but the broader psychosocial evidence remains insufficient to establish an effective intervention at this specific intersection [120].

## 5. Conclusions

Nutrition and body-image after SCI are connected in personal accounts, but remain distinct clinical and experiential domains. The 36-report map predominantly comprises qualitative evidence alongside a smaller quantitative literature combining exploratory associations and descriptive co-assessment. Participant accounts linked perceived weight or muscular change with acceptance, attractiveness, and participation, yet these accounts do not establish nutritional diagnoses, population prevalence, or causal pathways. Shared samples and uncertain statistics further limit independent confirmation. The proposed rehabilitation framework offers questions for coordinated assessment rather than a validated intervention. Progress requires repeated, appropriately measured outcomes in identifiable cohorts, clearer subgroup and denominator reporting, and broader representation of adolescents and underserved populations. Evaluating nutritional health and bodily experience separately will be essential to determining whether future interventions produce meaningful benefits in both.

## Figures and Tables

**Table 1:** Characteristics of the 36 included reports.

Report	Country and Setting	Design	Participants	Injury or Subgroup Information	Principal Eligible Contribution
Astorino and Harness, 2020 [51]	United States; community rehabilitation	Uncontrolled longitudinal ABT, 6 months	29 enrolled; 25 completers; 22 men/3 women; 18–59 years	12 tetraplegia/13 paraplegia; 9 complete/16 incomplete	Longitudinal change and exploratory association
Abedi et al., 2023 [52]	United States; rehabilitation outpatient clinic	Cross-sectional measurement-property study	106 adults (82 men); median age 45.5 years; 12 patient interviews within this sample; 6 professionals separately	SCI ≥ 3 months; median duration 13 years	Quantitative co-assessment
Rosito et al., 2002 [53]	United States; Palo Alto SCI service	Retrospective survey/chart series	27 respondents; 26 men/1 woman; 44–79 years	25 colostomies/2 ileostomies; long-standing SCI; one spouse proxy	Quantitative co-assessment
LaVela et al., 2021 [54]	United States; Chicago community	Thematic interviews, autumn 2018	25 adults; 24 men/1 woman; 26–83 years	11 veterans/14 civilians; 48% tetraplegia	Patient qualitative connection
LaVela et al., 2024 [55]	United States; national VHA	Descriptive qualitative interviews	33 veterans; 20 men/13 women; 29–84 years	18 tetraplegia/13 paraplegia/2 unknown; SCI ≥ 2 years	Patient qualitative connection
LaVela et al., 2024 [56]	United States; 11 VHA facilities	Descriptive qualitative interviews	12 professionals; 9 men/3 women; 26–64 years	9 dietitians, 2 physicians, 1 psychologist; no patient sample	Professional qualitative connection
Kehn and Kroll, 2009 [57]	United States; community	Exploratory telephone interviews	26 adults; 16 men/10 women; 23–74 years in Table 1	15 self-described exercisers/11 non-exercisers; SCI ≥ 1 year	Patient qualitative connection
Bailey et al., 2015 [58]	Canada; southern Ontario community	Modified grounded theory	9 adults; 4 men/5 women; 21–63 years	C3–T7; 4–36 years post-injury; shared cohort with 2016 report	Patient qualitative connection
Bailey et al., 2016 [59]	Canada; southern Ontario community	Modified grounded theory	9 adults; 4 men/5 women; 21–63 years	C3–T7; 4–36 years post-injury; same cohort as 2015 report	Patient qualitative connection
Bailey et al., 2018 [60]	Canada; Southern Ontario community follow-up	Qualitative focus group and individual interviews	6 unique participants; 3 men, 3 women; 23–68 years	C3–L3 SCI; 6–38 years after injury; subgroup of a previous dietary study	Patient qualitative connection
Kathnelson et al., 2020 [61]	Canada; community sample	Descriptive phenomenological interviews	6 men; 24–49 years	Complete/incomplete C4–T12 SCI; 7 months–29 years after injury	Patient qualitative connection
Chau et al., 2008 [62]	Canada; Ontario home interviews	Secondary qualitative analysis; modified grounded theory	15 women; 18–60 years	Traumatic paraplegia/tetraplegia; ≥3 years after initial rehabilitation; shared Ontario cohort	Patient qualitative connection
Sheldon et al., 2011 [63]	Canada; Ontario community	Secondary qualitative grounded-theory analysis	64 adult men from 80-person parent study	Acquired paraplegia/quadriplegia; ≥3 years after initial rehabilitation	Patient qualitative connection
Renwick et al., 2018 [64]	Canada; Ontario community sample	Secondary qualitative analysis; modified grounded theory	30 men; adult eligibility; current age range not reported	16 quadriplegia, 14 paraplegia; subgroup of the Ontario social-adaptation project	Patient qualitative connection
Barrett et al., 2023 [65]	United Kingdom; England community sample	Qualitative interviews; thematic analysis	20 adults; 15 male, 5 female; 25–65 years	Varied SCI levels/completeness; ≥18 months living in the community	Patient qualitative connection
Thrussell et al., 2018 [66]	United Kingdom; community, three SCI centers	Phenomenological interviews	27 women; 21–73 years in Table 1	Traumatic/non-traumatic SCI and CES; ≥1 year after rehabilitation	Patient qualitative connection
Martinsen et al., 2008 [67]	Denmark; home-based high-cervical SCI	Phenomenological interviews and meal observations	16 adults; 14 men/2 women; 18–65 years	Permanent assisted feeding; two interviews/person; 14 meal observations	Patient qualitative connection
Buchtler et al., 2021 [68]	Switzerland; post-acute rehabilitation	Qualitative pilot interviews	8 adults; 6 men/2 women; 19–62 years	Complete traumatic SCI; 2 tetraplegia/6 paraplegia	Patient qualitative connection
Desneves et al., 2026 [69]	Australia; acute and rehabilitation wards	Prospective mixed-methods implementation pilot	21 clinical patients; 12 interviewed (8 men, 4 women); 5 clinician focus-group participants	New traumatic SCI; clinical cohort median age 56 years; interview age distribution not separately reported	Patient qualitative connection
Beckwith and Yau, 2013 [70]	Australia; community interviews	Qualitative narrative study	7 women; 32–55 years	Acquired spinal injury; cervical to T12–L1; 4–39 years after injury	Patient qualitative connection
Parker and Yau, 2012 [71]	Australia; Queensland community sample	Qualitative interviews; thematic analysis	4 women; 31–53 years	Acquired SCI; 3 quadriplegia, 1 paraplegia; 15–30 years after injury	Patient qualitative connection
Kim et al., 2013 [72]	South Korea; community home visits	Cross-sectional survey and anthropometry	371 SCI participants; 308 men, 63 women; age 44.8 ± 9.6 years; body-perception AO subgroup 84	SCI > 1 year; 335 traumatic and 29 disease-related; variable injury characteristics	Quantitative co-assessment
Li and Yau, 2006 [73]	Hong Kong, China; home interviews	Qualitative narrative interviews	6 eligible SCI women, 32–44 years, within 10 participants; 4 poliomyelitis participants excluded	Adult-acquired SCI; 2 tetraplegia, 4 paraplegia; disability >2 years	Patient qualitative connection
Potgieter and Khan, 2005 [74]	South Africa; school-linked sample	Qualitative interviews with a vignette; thematic analysis	7 participants; 4 male, 3 female; 15–20 years	Acquired SCI; 4 paraplegia, 3 incomplete quadriplegia; ≥2 years after injury	Patient qualitative connection
Duarte Torres et al., 2016 [75]	Colombia; Bogotá rehabilitation-linked sample	Descriptive-interpretive qualitative interviews	12 participants; 8 men, 4 women; 2 adolescents and 10 young adults aged 20–24	Traumatic SCI ≥ 6 months; 9 thoracic, 2 cervical, 1 lumbar	Patient qualitative connection
Bassett and Martin Ginis, 2009 (men) [76]	Canada; SHAPE-SCI	Analytical cross-sectional	50 men; mean age 42.3 years; waist *n* = 49	25 paraplegia/25 tetraplegia; 20 complete/30 incomplete	Direct quantitative association
Bassett et al., 2009 (women) [77]	Canada; SHAPE-SCI	Analytical cross-sectional pilot	11 women; mean age 39.3 years	4 paraplegia/7 tetraplegia; 5 complete/6 incomplete	Direct quantitative association
Coggrave et al., 2012 [78]	United Kingdom; SCI ostomates	Retrospective cross-sectional postal survey	104 responses; 92 analyzed; 64 men/28 women; 24–86 years	26 cervical, 61 thoracic, 4 lumbar; 1 missing	Quantitative co-assessment
LaVela et al., 2021 (healthy eating) [79]	United States; veterans and civilians	Qualitative descriptive interviews	25 adults; 24 men/1 woman; 26–83 years	13 paraplegia/12 tetraplegia; possible linkage with weight-goal report	Patient qualitative belief
LaVela et al., 2025 [80]	United States; 11 VA hospitals	Qualitative provider interviews	12 professionals; 9 men/3 women; ages 26–64 years	9 dietitians, 2 physicians, 1 psychologist; not patient respondents	Professional qualitative link
Pellegrini et al., 2021 [81]	United States; VHA and regional SCI services	Qualitative descriptive provider interviews	25 professionals; 3 men/22 women; ages 26 to ≥65 years	Professional perspectives; patient demographics not sampled	Professional qualitative link
Perrier et al., 2014 [82]	North America; countries not separately itemized	Qualitative narrative analysis	SCI subgroup 9/11; full sample 7 women/4 men, 28–60 years	Adults with acquired disability; SCI-specific demographics incomplete	Patient qualitative link, SCI subgroup
Stensman, 1989 [83]	Sweden	Cross-sectional mixed-disability comparison	SCI subgroup 10/22; 8 men/2 women; 28–53 years	Traumatic tetraplegia; cerebral-palsy findings excluded	Quantitative co-assessment
Vinoski Thomas et al., 2019 [84]	United States	Qualitative mixed-disability interviews	Eligible SCI subgroup 5/15 women; 27–48 years	Other diagnoses excluded; one SCI participant also reported TBI	Patient qualitative link, SCI subgroup
Vázquez-Fariñas and Rodríguez-Martin, 2021 [85]	Spain; specialist SCI hospital	Qualitative grounded theory	32 adults; 17 men/15 women; 18–65 years	Acquired SCI; cervical, thoracic and lumbar; AIS A–D	Patient qualitative link
Watson et al., 2024 [86]	Australia; physically active adults	Analytical cross-sectional survey	105 adults; 66 men/39 women; mean age 56.1 years	Injury level, completeness and cause summaries conflict	Quantitative co-assessment

Abbreviations: SCI, spinal cord injury; SCI/D, spinal cord injuries and disorders; CES, cauda equina syndrome; AIS, American Spinal Injury Association Impairment Scale; VHA, Veterans Health Administration; NR, not reported. Sample sizes refer to the report or explicitly identified eligible subgroup, not unique participants across the review. Assessment age is distinguished from age at injury. Professional respondents are not patient samples.

**Table 2:** Nutritional and body-image constructs, findings, and forms of empirical contribution.

Report	Nutrition and Eating	Body-Image Construct	Finding	Evidence Contribution	Main Qualification
Astorino and Harness, 2020 [51]	DXA composition, waist and four-day dietary records	BSS and appearance component	Satisfaction improved while body fat increased. Published body-fat/ΔBSS association: r = 0.33, *p* = 0.049.	Longitudinal change and exploratory association	25 completers; no comparator. Correlation denominator/timing and reported r/*p* correspondence remain uncertain.
Abedi et al., 2023 [52]	No independent anthropometric or nutritional diagnosis; distinct IBS-QOL food-avoidance items retained	Separate body-image items retained; feeling-fat item removed	Item-set co-assessment only; no direct food-avoidance–BI association.	Quantitative co-assessment	106 patients; total alpha is not BI-domain validation, and structural fit remained limited.
Rosito et al., 2002 [53]	Recalled weight loss, diet and eating enjoyment	Study-specific body-image domain	Weight loss was reported by 8/27 before and 2/27 after stoma. Body-image scores were described as unchanged.	Quantitative co-assessment	Co-assessment without direct NI–BI test; retrospective recall and limited instrument validation.
LaVela et al., 2021 [54]	Weight-management targets and recalled weight	Appearance motivations and clothing fit	Appearance, health and mobility informed desired weight control and preferred feedback.	Patient qualitative connection	Goals and preferences, not achieved changes. Shares interviews with companion communication report.
LaVela et al., 2024 [55]	Perceived excess weight and recalled gain	Appearance concerns, clothing fit and mirror appraisal	Weight-related appearance concerns emerged alongside perceived physical and emotional burden.	Patient qualitative connection	Qualitative sample. The reported 42% is a coded theme frequency, not prevalence.
LaVela et al., 2024 [56]	Provider concerns about appetite suppression and eating adequacy	Perceived body-image vulnerability	Professionals linked concern about restrictive eating during pharmacotherapy with body-image vulnerability.	Professional qualitative connection	Provider perceptions only. No evidence here of drug-induced body-image harm or eating-disorder prevalence.
Kehn and Kroll, 2009 [57]	Recalled weight gain, loss and regain	Positive/negative appraisal of weight changes	Participants linked gained or regained weight with distress and weight loss with more positive feelings.	Patient qualitative connection	Subjective narratives within an exercise study; no quantified composition or direct association test.
Bailey et al., 2015 [58]	Eating as self-care, weight and bowel-management routines	Body acceptance, appreciation and gratitude	Dietary self-care and coordinated routines were described as supporting positive body experience.	Patient qualitative connection	Qualitative preliminary model; same nine-person cohort as Bailey 2016.
Bailey et al., 2016 [59]	Self-reported weight and perceived morphological change	Appearance, stigma and self-presentation	Weight and abdominal/muscular changes informed appearance concerns and strategies to manage others’ impressions.	Patient qualitative connection	Subjective accounts; shares nine-person cohort with Bailey 2015.
Bailey et al., 2018 [60]	Recalled weight loss; weight explicitly not measured Diet-adherence experiences, support, autonomy and access barriers	Appearance benefits and improved feelings about oneself in a focal account	Explicit qualitative diet/weight-loss–appearance link.	Patient qualitative connection	6 unique participants; selected qualitative follow-up does not estimate dietary efficacy.
Kathnelson et al., 2020 [61]	Perceived muscle loss, thinner limbs and abdominal enlargement No quantified intake	Appearance distress and concerns about attractiveness in intimate relationships	Explicit qualitative bodily change–appearance connection.	Patient qualitative connection	6 men; perceived muscular size is not measured muscle mass or diagnosed sarcopenia.
Chau et al., 2008 [62]	Perceived muscle/leg-shape and abdominal changes No quantified intake	Distress about clothing fit and mirror appearance, with evolving acceptance	Explicit qualitative connection between perceived bodily change and appearance evaluation	Patient qualitative connection	15 women from a shared parent cohort; perceived atrophy is not measured muscle loss.
Sheldon et al., 2011 [63]	Perceived thinness in a narrative	Appearance, clothing and self-concept	A participant linked feeling thin to concern about exposed ankles and clothing presentation.	Patient qualitative connection	Perceived morphology only; secondary analysis and shared parent cohort.
Renwick et al., 2018 [64]	Perceived overweight in a focal participant account No quantified intake	Feeling better about appearance and managing public staring	Narrative connection between perceived weight and positive self-presentation.	Patient qualitative connection	30 men from an overlapping parent cohort; focal account is not a prevalence estimate.
Barrett et al., 2023 [65]	One participant recalled gain, abdominal enlargement and subsequent weight loss No quantified intake	Clothed-body confidence improved after weight loss, while unclothed discomfort persisted	Qualitative weight-change–body-confidence account.	Patient qualitative connection	20 participants; the focal account is not a measured trajectory or population frequency.
Thrussell et al., 2018 [66]	Recalled weight/shape changes; food and fluid avoidance	Body acceptance and sexual self-image	Weight gain was linked to appearance dissatisfaction and avoidance of mirrors or photographs.	Patient qualitative connection	Qualitative accounts; eating avoidance for continence and appearance concerns are distinct motives.
Martinsen et al., 2008 [67]	Assisted eating, food avoidance and meal devices	Embodied self-image and meal-related presentation	Food/device choices and the manner of assistance were negotiated to preserve dignity and recognizable self-presentation.	Patient qualitative connection	No quantified intake or nutrition–image association;16 persons, despite repeated interviews.
Buchtler et al., 2021 [68]	Subjectively smaller muscles	Body experience, appearance and self-recognition	Perceived muscle-size reduction could coexist with continuity in body identity.	Patient qualitative connection	Individual qualitative narrative; no composition measurement or nutritional diagnosis.
Desneves et al., 2026 [69]	BIS/anthropometry in 21 clinical patients; interview accounts of weight and muscle changes; dietary quantity and protein choices discussed after composition feedback	Unwanted weight/shape concerns and encouraging interpretation of muscle gain	Qualitative connection between composition feedback, eating and self-image.	Patient qualitative connection	12 patient interviews; clinical measurements are contextual, without a composition–BI association test.
Beckwith and Yau, 2013 [70]	Perceived muscle loss, excess weight and abdominal change No quantified intake	Appearance distress, femininity and evolving body acceptance	Explicit qualitative link between bodily change descriptions and appearance evaluation.	Patient qualitative connection	7 women; perceived fat/muscle changes are not objective composition outcomes.
Parker and Yau, 2012 [71]	Perceived abdominal enlargement and absent musculature No quantified intake	Dislike of stomach and changed body/sexual self-image	Explicit qualitative descriptor–appearance-evaluation link.	Patient qualitative connection	4 women; abdominal appearance is not measured adiposity.
Kim et al., 2013 [72]	Measured waist classification; AO perception subgroup 84 45/84 (53.6%) reported dietary modification for weight control	42/84 (50.0%) perceived themselves as fat/very fat	Measured-size and perceived-size comparison; no adjusted BI association estimate.	Quantitative co-assessment	371 enrolled; analysis denominators inconsistent. Perceived size is not body dissatisfaction.
Li and Yau, 2006 [73]	Perceived leg thinning and abdominal/chin size in SCI-attributable accounts No quantified intake	Appearance embarrassment, unattractiveness and concealment	Explicit qualitative bodily change–appearance links in identifiable SCI narratives.	Patient qualitative connection	Only 6 SCI women are eligible; whole-sample frequencies are not SCI-specific.
Potgieter and Khan, 2005 [74]	Recalled weight loss and perceived thinness; no anthropometry Dieting described as a personal self-care practice	Attractiveness concerns, body acceptance and sexual self-esteem	Explicit narrative link between weight/thinness and appearance evaluation.	Patient qualitative connection	7 participants; no nutritional diagnosis, effect estimate or prevalence inference.
Duarte Torres et al., 2016 [75]	Retrospectively reported major weight loss and changed appearance Hospital-food dissatisfaction/rejection described; no quantified intake	Transformed self-image and psychological distress	Explicit qualitative weight-change–self-image account.	Patient qualitative connection	12 participants; only 2 adolescents; recalled weight is not a verified trajectory.
Bassett and Martin Ginis, 2009 (men) [76]	Measured weight, waist circumference and BIA-derived body fat	Adult Body Satisfaction Questionnaire, appearance and function	Appearance r = 0.08 for weight, 0.21 for waist and 0.00 for body fat. Functional r = 0.22, 0.21 and 0.08, respectively. All were reported nonsignificant.	Direct quantitative association	n = 50, except waist n = 49. Unadjusted cross-sectional correlations without confidence intervals.
Bassett et al., 2009 (women) [77]	Measured weight, waist circumference and BIA-derived body fat	Adult Body Satisfaction Questionnaire, appearance and function	Appearance–waistρ = −0.64, *p* < 0.05; appearance–body fatρ = −0.46, *p* < 0.10; appearance–weightρ = −0.33, not significant.	Direct quantitative association	n = 11; one-tailed Spearman tests without adjustment. Functional correlations were not significant.
Coggrave et al., 2012 [78]	Self-reported dietary manipulation for bowel care	Study-specific appearance/stoma questions and TSCS physical self	Dietary manipulation reportedly decreased after stoma formation, *p* = 0.001; 33% reported appearance change.	Quantitative co-assessment	Retrospective self-report without an NI–BI test. Published body-image counts and TSCS summary labels contain inconsistencies.
LaVela et al., 2021 (healthy eating) [79]	Personal meanings of healthy eating	Desired skin appearance in a participant account	One participant linked healthy eating with desired skin appearance.	Patient qualitative belief	Peripheral appearance-related evidence, without a global body-image assessment or measured benefit.
LaVela et al., 2025 [80]	Provider accounts of diet, weight and body-composition changes	Provider-described body-image concerns	Accounts linked poor diet, perceived muscle loss or fat gain, and body-image concerns.	Professional qualitative link	Professional perspectives, not measured patient associations. Causal direction remained uncertain.
Pellegrini et al., 2021 [81]	Weight-management behaviors, barriers and facilitators	Body-image concerns as a psychological barrier	Providers identified body-image concerns among psychological barriers to weight management.	Professional qualitative link	Thematic professional appraisal, not a patient-level association.
Perrier et al., 2014 [82]	Recalled leg-muscle loss	Visual and emotional appraisal of changed muscular appearance	Perceived visible leg-muscle loss was connected in one participant account with sadness about bodily change.	Patient qualitative link, SCI subgroup	Muscularity description and negative body appraisal were distinct. Athletic identity alone is insufficient body-image evidence.
Stensman, 1989 [83]	BMI calculated from actual weight and height	Situation-specific/global VAS ratings and Body Puzzle estimates	Global body-image VAS mean 57.2/100 in the SCI subgroup; BMI and body-size estimates were also reported.	Quantitative co-assessment	SCI subgroup n = 10; no NI–BI association test.
Vinoski Thomas et al., 2019 [84]	Recalled weight and muscle/body-size changes	Appearance and functional-esthetic body-image	Recalled weight gain and muscle loss were linked with dislike or distress about appearance.	Patient qualitative link, SCI subgroup	Five eligible SCI participants. No objective weight-change or NI–BI association estimate. Non-SCI eating accounts were not extracted.
Vázquez-Fariñas and Rodríguez-Martin, 2021 [85]	Perceived muscle loss and leg thinning	Appearance appraisal, body recognition and avoidance	Perceived leg thinning and muscle loss were connected with negative appearance appraisals and shorts avoidance.	Patient qualitative link	Subjective bodily descriptors, not diagnostic evidence of sarcopenia.
Watson et al., 2024 [86]	Perceived weight-management gains, EMGI	Perceived appearance gains, EMGI	Separate perceived weight-management and appearance gains were reported.	Quantitative co-assessment	Not measured weight loss or a direct NI–BI association. Pooled/subgroup means are internally inconsistent.

Abbreviations: SCI, spinal cord injury; BIA, bioelectrical impedance analysis; BIS, bioimpedance spectroscopy; BMI, body mass index; BSS, Body Satisfaction Survey; BSS-A, appearance component; DXA, dual-energy X-ray absorptiometry; IBS-QOL, Irritable Bowel Syndrome Quality of Life Questionnaire; NR, not reported; VAS, visual analog scale; Δ, change. Association denotes a direct analysis, co-assessment denotes separate constructs measured in the same sample without a direct test, and qualitative connection denotes an explicit empirical account. Perceived morphology and recalled change are not objective composition measurements.

**Table 3:** Thematic findings, evidential limits, and research priorities.

Thematic Domain	Contributing Evidence	Mapped Finding	Evidential Limit	Conceptual Assessment Question	Research Priority
Composition and bodily evaluation	Astorino [51]; SHAPE-SCI [76,77]	Composition and satisfaction can differ; exploratory correlations are inconsistent.	Uncontrolled change, small samples and uncertain statistics.	Which clinical and experiential outcomes matter separately?	Repeated linked measurements with confounder control and explicit denominators.
Perceived size and appearance	Kim [72]; Stensman [83]; patient accounts	Perceived size and measured classification do not necessarily correspond.	Perceived size is not equivalent to dissatisfaction or diagnosis.	Does the concern involve size, attractiveness, comfort, or health?	Validate distinct size-perception and evaluative measures in SCI.
Muscularity, identity and acceptance	Buchtler [68]; Vázquez-Fariñas [85]; Perrier [82]	Perceived thinning or muscle loss can affect recognition, clothing and attractiveness.	Subjective descriptions do not establish measured tissue loss or sarcopenia.	What does the change mean for the person?	Combine appropriate composition assessment with individual qualitative trajectories.
Eating, bodily presentation and control	Martinsen [67]; Bailey [58,60]; LaVela [54,55]	Food, assistance and weight goals acquire meaning through dignity and appearance.	Restriction motives vary; recalled benefits do not establish nutritional adequacy.	Do symptoms, assistance, access or appearance concerns shape eating?	Measure intake and explicit motives alongside distinct body-image outcomes.
Bowel care and body-image	Rosito [53]; Abedi [52]; Coggrave [78]	Dietary, weight and body-image domains are assessed alongside bowel experiences.	Co-assessment, retrospective recall, and uncertain domain properties.	Which eating and bodily concerns remain after bowel-related change?	Prospective domain-specific assessment with appropriate measurement validation.
Professional interpretations	LaVela [56,80]; Pellegrini [81]	Providers describe psychological barriers and perceived nutritional/body-image links.	Provider accounts are not patient prevalence or causal evidence; possible sample overlap.	Which professional assumptions correspond to patient priorities?	Compare patient and provider accounts within clearly identified samples.
Development and representation	Potgieter [74]; Duarte [75]; sex-specific reports	Adolescent and young-adult accounts highlight thinness and attractiveness concerns.	Small samples, age inconsistencies and limited developmental diversity.	How do age, gendered expectations, and context shape the concern?	Age-specific measures, diverse recruitment, and explicit subgroup reporting.
Coordinated rehabilitation	Synthesis of the mapped domains	Distinct nutritional, experiential and participation goals may be considered together.	The combined framework has not been evaluated as an intervention.	Can a feasible plan address the person’s separate priorities?	Co-designed feasibility and outcome studies before standardized implementation.

Themes can share reports and participants. Clinical considerations are conceptual interpretations requiring evaluation, not demonstrated treatment effects. Professional accounts remain distinct from patient experiences. Appraisal did not determine inclusion through a numerical threshold. Abbreviations: SCI, spinal cord injury; BMI, body mass index; BSS, Body Satisfaction Survey; DXA, dual-energy X-ray absorptiometry.

## Data Availability

The charted report characteristics, findings, and methodological appraisals are provided in the article and Supplementary Materials. The protocol is available through the Open Science Framework project XCBHR: https://doi.org/10.17605/OSF.IO/XCBHR. Primary publications are identified in the reference list. Report linkage and analytical denominators are distinguished from independent participant counts.

## Supplementary Materials

The following supporting information can be downloaded at https://www.mdpi.com/article/10.3390/nu18213604/s1. Table S1: Review design, protocol, search chronology and responsibilities; Table S2: Population, Concept and Context framework and eligibility boundaries; Table S3: Information sources, executable search expressions and search documentation; Table S4: Selection procedures and reconciled PRISMA counts; Table S5: Data charting and evidence synthesis procedures; Table S6: Detailed report findings, analytical denominators and contribution-specific limitations; Table S7: Methodological appraisal by report and empirical contribution; Table S8: Cohort linkage, subgroup attribution and report counts; Table S9: Measurement limitations, reporting inconsistencies and research priorities; Table S10: Conceptual applications of the evidence map to coordinated rehabilitation assessment.

## References

[1] Ahuja C.S., Wilson J.R., Nori S., Kotter M.R.N., Druschel C., Curt A., Fehlings M.G. (2017). Traumatic spinal cord injury. Nat. Rev. Dis. Primers.

[2] (2019). GBD 2016 Traumatic Brain Injury and Spinal Cord Injury Collaborators. Global, regional, and national burden of traumatic brain injury and spinal cord injury, 1990–2016: A systematic analysis for the Global Burden of Disease Study 2016. Lancet Neurol..

[3] Sezer N., Akkuş S., Uğurlu F.G. (2015). Chronic complications of spinal cord injury. World J. Orthop..

[4] Farkas G.J., Gater D.R. (2018). Neurogenic obesity and systemic inflammation following spinal cord injury: A review. J. Spinal Cord Med..

[5] Li Z., Wang X., Yu Y., Jing Y., Du H., Liu W., Zhang C., Talifu Z., Xu X., Pan Y. (2025). Nutritional alterations, adverse consequences, and comprehensive assessment in spinal cord injury: A review. Front. Nutr..

[6] Nash M.S., Groah S.L., Gater D.R., Dyson-Hudson T.A., Lieberman J.A., Myers J., Sabharwal S., Taylor A.J., Consortium for Spinal Cord Medicine (2018). Identification and Management of Cardiometabolic Risk After Spinal Cord Injury: Clinical Practice Guideline for Health Care Providers. Top. Spinal Cord Inj. Rehabil..

[7] Cederholm T., Barazzoni R., Austin P., Ballmer P., Biolo G., Bischoff S.C., Compher C., Correia I., Higashiguchi T., Holst M. (2017). ESPEN guidelines on definitions and terminology of clinical nutrition. Clin. Nutr..

[8] Cederholm T., Jensen G.L., Correia M.I.T.D., Gonzalez M.C., Fukushima R., Higashiguchi T., Baptista G., Barazzoni R., Blaauw R., Coats A. (2019). GLIM criteria for the diagnosis of malnutrition—A consensus report from the global clinical nutrition community. Clin. Nutr..

[9] Cederholm T., Jensen G.L., Correia M.I.T.D., Gonzalez M.C., Fukushima R., Pisprasert V., Blaauw R., Braz D.C., Carrasco F., Cruz Jentoft A.J. (2025). The GLIM consensus approach to diagnosis of malnutrition: A 5-year update. Clin. Nutr..

[10] Farkas G.J., Pitot M.A., Berg A.S., Gater D.R. (2019). Nutritional status in chronic spinal cord injury: A systematic review and meta-analysis. Spinal Cord.

[11] Raguindin P.F., Bertolo A., Zeh R.M., Fränkl G., Itodo O.A., Capossela S., Bally L., Minder B., Brach M., Eriks-Hoogland I. (2021). Body Composition According to Spinal Cord Injury Level: A Systematic Review and Meta-Analysis. J. Clin. Med..

[12] van der Scheer J.W., Totosy de Zepetnek J.O., Blauwet C., Brooke-Wavell K., Graham-Paulson T., Leonard A.N., Webborn N., Goosey-Tolfrey V.L. (2021). Assessment of body composition in spinal cord injury: A scoping review. PLoS ONE.

[13] Laughton G.E., Buchholz A.C., Martin Ginis K.A., Goy R.E., SHAPE SCI Research Group (2009). Lowering body mass index cutoffs better identifies obese persons with spinal cord injury. Spinal Cord.

[14] Cruz-Jentoft A.J., Bahat G., Bauer J., Boirie Y., Bruyère O., Cederholm T., Cooper C., Landi F., Rolland Y., Sayer A.A. (2019). Sarcopenia: Revised European consensus on definition and diagnosis. Age Ageing.

[15] Donini L.M., Busetto L., Bischoff S.C., Cederholm T., Ballesteros-Pomar M.D., Batsis J.A., Bauer J.M., Boirie Y., Cruz-Jentoft A.J., Dicker D. (2022). Definition and diagnostic criteria for sarcopenic obesity: ESPEN and EASO consensus statement. Clin. Nutr..

[16] Farkas G.J., Sneij A., McMillan D.W., Tiozzo E., Nash M.S., Gater D.R. (2022). Energy expenditure and nutrient intake after spinal cord injury: A comprehensive review and practical recommendations. Br. J. Nutr..

[17] McMillan D.W., Bigford G.E., Farkas G.J. (2023). The Physiology of Neurogenic Obesity: Lessons from Spinal Cord Injury Research. Obes. Facts.

[18] Gater D.R., Bauman C., Cowan R. (2020). A Primary Care Provider’s Guide to Diet and Nutrition After Spinal Cord Injury. Top. Spinal Cord Inj. Rehabil..

[19] van Strien T. (2018). Causes of Emotional Eating and Matched Treatment of Obesity. Curr. Diab. Rep..

[20] Stunkard A.J., Messick S. (1985). The three-factor eating questionnaire to measure dietary restraint, disinhibition and hunger. J. Psychosom. Res..

[21] Tylka T.L., Kroon Van Diest A.M. (2013). The Intuitive Eating Scale-2: Item refinement and psychometric evaluation with college women and men. J. Couns. Psychol..

[22] Tylka T.L., Wood-Barcalow N.L. (2015). What is and what is not positive body image? Conceptual foundations and construct definition. Body Image.

[23] Webb J.B., Wood-Barcalow N.L., Tylka T.L. (2015). Assessing positive body image: Contemporary approaches and future directions. Body Image.

[24] Tylka T.L., Wood-Barcalow N.L. (2015). The Body Appreciation Scale-2: Item refinement and psychometric evaluation. Body Image.

[25] Alleva J.M., Tylka T.L. (2021). Body functionality: A review of the literature. Body Image.

[26] Alleva J.M., Tylka T.L., Kroon Van Diest A.M. (2017). The Functionality Appreciation Scale (FAS): Development and psychometric evaluation in U.S. community women and men. Body Image.

[27] Mehling W.E., Price C., Daubenmier J.J., Acree M., Bartmess E., Stewart A. (2012). The Multidimensional Assessment of Interoceptive Awareness (MAIA). PLoS ONE.

[28] Garfinkel S.N., Seth A.K., Barrett A.B., Suzuki K., Critchley H.D. (2015). Knowing your own heart: Distinguishing interoceptive accuracy from interoceptive awareness. Biol. Psychol..

[29] Nash A., Kaiser S., Ledwith S. (2024). Individual’s experiences of adjusting to changes in body image after spinal cord injury: A literature review and thematic synthesis. Disabil. Rehabil..

[30] Cunha I.M., Nett S., Jarvie A., Rodgers R.F. (2026). Positive body image and chronic illness: A systematic review. J. Health Psychol..

[31] Raffman E., Jayabalan P. (2025). Disordered Eating Behaviors in Individuals with Physical Disabilities: A Narrative Review. Am. J. Lifestyle Med..

[32] Bonder R., Walker M., Toulany A., McPherson A.C. (2025). What Is Known About Eating Disorders and Disordered Eating in Individuals with Physical Disabilities? A Narrative Review. Child Care Health Dev..

[33] Madigan C.D., King J.A., Taylor C., Hoekstra S.P., Graham H.E., Kirk N., Fenton J.M., Goosey-Tolfrey V.L. (2024). A systematic review and qualitative synthesis of weight management interventions for people with spinal cord injury. Obes. Rev..

[34] Chieh C., Stojic S., Boehl G., Wong S., Lüscher J., Bertolo A., Itodo O.A., Mueller G., Stoyanov J., Gemperli A. (2025). Can Lifestyle and Behavioral Interventions Improve Weight Management in Individuals with Spinal Cord Injury? A Systematic Review and Meta-analysis. Arch. Phys. Med. Rehabil..

[35] Stojic S., Eriks-Hoogland I., Gamba M., Valido E., Minder B., Chatelan A., Karagounis L.G., Ballesteros M., Díaz C., Brach M. (2023). Mapping of Dietary Interventions Beneficial in the Prevention of Secondary Health Conditions in Spinal Cord Injured Population: A Systematic Review. J. Nutr. Health Aging.

[36] Munn Z., Peters M.D.J., Stern C., Tufanaru C., McArthur A., Aromataris E. (2018). Systematic review or scoping review? Guidance for authors when choosing between a systematic or scoping review approach. BMC Med. Res. Methodol..

[37] Peters M.D.J., Marnie C., Tricco A.C., Pollock D., Munn Z., Alexander L., McInerney P., Godfrey C.M., Khalil H. (2020). Updated methodological guidance for the conduct of scoping reviews. JBI Evid. Synth..

[38] Peters M.D.J., Godfrey C., McInerney P., Khalil H., Larsen P., Marnie C., Pollock D., Tricco A.C., Munn Z. (2022). Best practice guidance and reporting items for the development of scoping review protocols. JBI Evid. Synth..

[39] Tricco A.C., Lillie E., Zarin W., O’Brien K.K., Colquhoun H., Levac D., Moher D., Peters M.D.J., Horsley T., Weeks L. (2018). PRISMA Extension for Scoping Reviews (PRISMA-ScR): Checklist and Explanation. Ann. Intern. Med..

[40] Aromataris E., Lockwood C., Porritt K., Pilla B., Jordan Z. (2024). JBI Manual for Evidence Synthesis.

[41] Rethlefsen M.L., Kirtley S., Waffenschmidt S., Ayala A.P., Moher D., Page M.J., Koffel J.B., PRISMA-S Group (2021). PRISMA-S: An extension to the PRISMA Statement for Reporting Literature Searches in Systematic Reviews. Syst. Rev..

[42] McGowan J., Sampson M., Salzwedel D.M., Cogo E., Foerster V., Lefebvre C. (2016). PRESS Peer Review of Electronic Search Strategies: 2015 Guideline Statement. J. Clin. Epidemiol..

[43] Bramer W.M., Giustini D., de Jonge G.B., Holland L., Bekhuis T. (2016). De-duplication of database search results for systematic reviews in EndNote. J. Med. Libr. Assoc..

[44] Pollock D., Peters M.D.J., Khalil H., McInerney P., Alexander L., Tricco A.C., Evans C., de Moraes É.B., Godfrey C.M., Pieper D. (2023). Recommendations for the extraction, analysis, and presentation of results in scoping reviews. JBI Evid. Synth..

[45] Mokkink L.B., Elsman E.B.M., Terwee C.B. (2024). COSMIN guideline for systematic reviews of patient-reported outcome measures version 2.0. Qual. Life Res..

[46] Moola S., Munn Z., Tufanaru C., Aromataris E., Sears K., Sfetcu R., Currie M., Lisy K., Qureshi R., Mattis P., Aromataris E., Munn Z. (2020). Chapter 7: Systematic reviews of etiology and risk. JBI Manual for Evidence Synthesis.

[47] Barker T.H., Habibi N., Aromataris E., Stone J.C., Leonardi-Bee J., Sears K., Hasanoff S., Klugar M., Tufanaru C., Moola S. (2024). The revised JBI critical appraisal tool for the assessment of risk of bias for quasi-experimental studies. JBI Evid. Synth..

[48] Hsieh H.F., Shannon S.E. (2005). Three approaches to qualitative content analysis. Qual. Health Res..

[49] Page M.J., McKenzie J.E., Bossuyt P.M., Boutron I., Hoffmann T.C., Mulrow C.D., Shamseer L., Tetzlaff J.M., Akl E.A., Brennan S.E. (2021). The PRISMA 2020 statement: An updated guideline for reporting systematic reviews. BMJ.

[50] Khalil H., Peters M.D., Tricco A.C., Pollock D., Alexander L., McInerney P., Godfrey C.M., Munn Z. (2021). Conducting high quality scoping reviews-challenges and solutions. J. Clin. Epidemiol..

[51] Astorino T.A., Harness E.T. (2020). Improved quality of life and body satisfaction in response to activity-based therapy in adults with spinal cord injury. Neuroimmunol. Neuroinflamm..

[52] Abedi A., Kohli P., Montero S., Morales L., Ha N., Abedi A., La Riva A., Ginsberg D., Kreydin E. (2023). Content, face, and construct validity of the Irritable Bowel Syndrome Quality of Life (IBS-QOL) as a measure of bowel-related quality of life in spinal cord injury. Neurourol. Urodyn..

[53] Rosito O., Nino-Murcia M., Wolfe V.A., Kiratli B.J., Perkash I. (2002). The effects of colostomy on the quality of life in patients with spinal cord injury: A retrospective analysis. J. Spinal Cord Med..

[54] LaVela S.L., Jones K., Heinemann A.W., Bartle B., Ehrlich-Jones L.S. (2021). Motivators, goal setting, and helpful feedback for weight management among individuals with spinal cord injury. Rehabil. Psychol..

[55] LaVela S.L., Wu J., Harris A.H.S., Frayne S.M., Nevedal A.L., Arnow K.D., Barreto N.B., Davis K., Eisenberg D. (2024). Physical and emotional consequences of excess weight as experienced by individuals with spinal cord injuries. J. Spinal Cord Med..

[56] LaVela S.L., Berryman K., Kale I., Farkas G.J., Henderson G.V., Rosales V., Eisenberg D., Reyes L. (2024). Potential barriers to the use of anti-obesity medications in persons with spinal cord injuries and disorders. Obes. Sci. Pract..

[57] Kehn M., Kroll T. (2009). Staying physically active after spinal cord injury: A qualitative exploration of barriers and facilitators to exercise participation. BMC Public Health.

[58] Bailey K.A., Gammage K.L., van Ingen C., Ditor D.S. (2015). “It’s all about acceptance”: A qualitative study exploring a model of positive body image for people with spinal cord injury. Body Image.

[59] Bailey K.A., Gammage K.L., van Ingen C., Ditor D.S. (2016). Managing the stigma: Exploring body image experiences and self-presentation among people with spinal cord injury. Health Psychol. Open.

[60] Bailey K.A., Lenz K., Allison D.J., Ditor D.S. (2018). Barriers and facilitators to adhering to an anti-inflammatory diet for individuals with spinal cord injuries. Health Psychol. Open.

[61] Kathnelson J.D., Kurtz Landy C.M., Ditor D.S., Tamim H., Gage W.H. (2020). Examining the psychological and emotional experience of sexuality for men after spinal cord injury. Cogent Psychol..

[62] Chau L., Hegedus L., Praamsma M., Smith K., Tsukada M., Yoshida K., Renwick R. (2008). Women living with a spinal cord injury: Perceptions about their changed bodies. Qual. Health Res..

[63] Sheldon A.P., Renwick R., Yoshida K.K. (2011). Exploring body image and self-concept of men with acquired spinal cord injuries. Am. J. Mens Health.

[64] Renwick R., Yoshida K., Eacrett E., Rose N. (2018). Meaning of Staring and the Starer-Staree Relationship Related to Men Living with Acquired Spinal Cord Injuries. Am. J. Mens Health.

[65] Barrett O.E.C., Mattacola E., Finlay K.A. (2023). “You feel a bit unsexy sometimes”: The psychosocial impact of a spinal cord injury on sexual function and sexual satisfaction. Spinal Cord.

[66] Thrussell H., Coggrave M., Graham A., Gall A., Donald M., Kulshrestha R., Geddis T. (2018). Women’s experiences of sexuality after spinal cord injury: A UK perspective. Spinal Cord.

[67] Martinsen B., Harder I., Biering-Sorensen F. (2008). The meaning of assisted feeding for people living with spinal cord injury: A phenomenological study. J. Adv. Nurs..

[68] Buchtler K., Carrard V., Jochum H., Ledermann K., Lude P., Scheel-Sailer A. (2021). Body experience during post-acute rehabilitation in individuals after a traumatic spinal cord injury: A qualitative interview-based pilot study. Spinal Cord Ser. Cases.

[69] Desneves K.J., Fittall B., Elson C., Daly R.M., Ward L.C., Kiss N. (2026). Implementing body composition assessment into clinical practice in patients with acute spinal cord injury- a pilot feasibility study. Spinal Cord.

[70] Beckwith A., Yau M.K.-S. (2013). Sexual recovery: Experiences of women with spinal injury reconstructing a positive sexual identity. Sex. Disabil..

[71] Parker M.G., Yau M.K. (2012). Sexuality, identity and women with spinal cord injury. Sex. Disabil..

[72] Kim K.D., Nam H.S., Shin H.I. (2013). Characteristics of abdominal obesity in persons with spinal cord injury. Ann. Rehabil. Med..

[73] Li C.M.-N., Yau M.K.-S. (2006). Sexual issues and concerns: Tales of Chinese women with spinal cord impairments. Sex. Disabil..

[74] Potgieter C.-A., Khan G. (2005). Sexual self-esteem and body image of South African spinal cord injured adolescents. Sex. Disabil..

[75] Duarte Torres D.M., Torres Bolaños Y.M., Moreno Fergusson M.E. (2016). Body and Corporality in adolescents and young adults with spinal cord injury. Investig. Educ. Enferm..

[76] Bassett R.L., Martin Ginis K.A., SHAPE-SCI Research Group (2009). More than looking good: Impact on quality of life moderates the relationship between functional body image and physical activity in men with SCI. Spinal Cord.

[77] Bassett R.L., Martin Ginis K.A., Buchholz A.C., SHAPE SCI Research Group (2009). A pilot study examining correlates of body image among women living with SCI. Spinal Cord.

[78] Coggrave M.J., Ingram R.M., Gardner B.P., Norton C.S. (2012). The impact of stoma for bowel management after spinal cord injury. Spinal Cord.

[79] LaVela S.L., Ehrlich-Jones L.S., Jones K., Bartle B., Heinemann A.W. (2021). What Are Personal Meanings of Healthy Eating and Physical Activity in Individuals Living with Spinal Cord Injury? A Qualitative Exploration. Top. Spinal Cord Inj. Rehabil..

[80] LaVela S.L., Farkas G.J., Berryman K., Kale I.O., Sneij A., Felix E.R., Reyes L. (2025). Health consequences associated with poor diet and nutrition in persons with spinal cord injuries and disorders. Disabil. Rehabil..

[81] Pellegrini C.A., Burkhart L., Jones K., LaVela S.L. (2021). Health provider-identified barriers and facilitators to weight management for individuals with spinal cord injury. Spinal Cord.

[82] Perrier M.J., Smith B., Strachan S.M., Latimer-Cheung A.E. (2014). Narratives of athletic identity after acquiring a permanent physical disability. Adapt. Phys. Act. Q..

[83] Stensman R. (1989). Body image among 22 persons with acquired and congenital severe mobility impairment. Paraplegia.

[84] Vinoski Thomas E., Warren-Findlow J., Webb J.B., Quinlan M.M., Laditka S.B., Reeve C.L. (2019). “It’s very valuable to me that I appear capable”: A qualitative study exploring relationships between body functionality and appearance among women with visible physical disabilities. Body Image.

[85] Vázquez-Fariñas M., Rodríguez-Martin B. (2021). “Living with a fragmented body”: A qualitative study on perceptions about body changes after a spinal cord injury. Spinal Cord.

[86] Watson P.K., Stendell L., Quel de Oliveira C., Middleton J.W., Arora M., Davis G.M. (2024). Leisure-time physical activity motives and perceived gains for individuals with spinal cord injury. Spinal Cord.

[87] LaVela S.L., Wu J., Nevedal A.L., Frayne S.M., Harris A.H.S., Arnow K.D., Davis K., Farkas G.J., Reyes L., Eisenberg D. (2025). Nutrition and eating beliefs and behaviors among individuals with spinal cord injuries and disorders: Healthy or misconceived?. Rehabil. Psychol..

[88] Li Z., Wang X., Du H., Liu W., Zhang C., Talifu Z., Xu X., Pan Y., Zhang J., Ke H. (2025). Unraveling Spinal Cord Injury Nutrition: Effects of Diet on the Host and Microbiome. Adv. Nutr..

[89] Areni A., Capeci W., Cassinis A., De Palma L., Del Popolo G., Fergnani F., Pelizzari L. (2024). What We Do and What We Should Do Against Malnutrition in Spinal Cord Injury: A Position Paper From Italian Spinal Cord Injury Network Rehabilitation Centers. J. Clin. Med. Res..

[90] Zengul A.G., Ferguson C.C., Rimmer J.H., Cofield S.S., Davis E.N., Hill J.O., Thirumalai M. (2025). Expert-Reviewed Nutritional Guidance for Adults with Spinal Cord Injury: A Delphi Study. Nutrients.

[91] Flury I., Mueller G., Perret C. (2023). The risk of malnutrition in patients with spinal cord injury during inpatient rehabilitation-A longitudinal cohort study. Front. Nutr..

[92] Tamai K., Terai H., Nakamura H., Yokogawa N., Sasagawa T., Nakashima H., Segi N., Ito S., Funayama T., Eto F. (2024). Impact of malnutrition on mortality and neurological recovery of older patients with spinal cord injury. Sci. Rep..

[93] Desneves K.J., Kiss N., Daly R.M., Abbott G., Ward L.C. (2024). Longitudinal changes in body composition and diet after acute spinal cord injury. Nutrition.

[94] Nevin A.N., Atresh S.S., Vivanti A., Ward L.C., Hickman I.J. (2024). Resting energy expenditure during spinal cord injury rehabilitation and utility of fat-free mass-based energy prediction equations: A pilot study. Spinal Cord Ser. Cases.

[95] Faber W.X.M., Nachtegaal J., Stolwijk J., Witteman B.J.M., Winkels R. (2025). Influence of Dietary Quality on Neurogenic Bowel Dysfunction in Individuals with Spinal Cord Injury. Top. Spinal Cord Inj. Rehabil..

[96] Nevin A.N., Dwyer K., Atresh S.S., Vivanti A., Hickman I.J., Banks M. (2025). Nutritional Intakes of People with Spinal Cord Injury Who Have Pressure Injuries in Hospital: A Secondary Analysis of a Randomized Controlled Trial. Top. Spinal Cord Inj. Rehabil..

[97] Slettahjell H.B., Strøm V., Thoresen M., Strøm M., Glott T., Biering-Sørensen F., Henriksen C. (2026). Effects of an individualized dietitian-led nutrition intervention on body composition during the first year after traumatic spinal cord injury: A randomized controlled trial. Clin. Nutr. ESPEN.

[98] Froehlich-Grobe K. (2024). Self-Reported Difficulty with and Assistance Needed by People with Spinal Cord Injury to Prepare Meals at Home. Int. J. Environ. Res. Public Health.

[99] Donato Z.J., Levy A.S., Sneij A., LaVela S.L., Berg A.S., Shapiro L.T., Farkas G.J. (2025). Grocery Shopping and Eating Behaviors Differ Between Persons with and Without a Spinal Cord Injury. Top. Spinal Cord Inj. Rehabil..

[100] Li J., Wen H., Yarar-Fisher C., Chen Y. (2025). Evaluating Racial Disparities in Food Access and Their Impact on Body Mass Index Among Individuals with Traumatic Spinal Cord Injury. Arch. Phys. Med. Rehabil..

[101] Farkas G.J., Cunningham P.M., Sneij A.M., Hayes J.E., Nash M.S., Berg A.S., Gater D.R., Rolls B.J. (2024). Reasons for meal termination, eating frequency, and typical meal context differ between persons with and without a spinal cord injury. Appetite.

[102] Widaman A., Dirlikov B., Gopalan R., Wood S., Casas M.S., Shem K. (2026). Development of Telenutrition Education and Counseling Protocol to Improve Diet Quality in Individuals with Spinal Cord Injury. Top. Spinal Cord Inj. Rehabil..

[103] Li J., Gower B., McLain A., Yarar-Fisher C. (2022). Effects of a low-carbohydrate/high-protein diet on metabolic health in individuals with chronic spinal cord injury: An exploratory analysis of results from a randomized controlled trial. Physiol. Rep..

[104] Li J., Morrow C., McLain A., Womack E.D., Yarar-Fisher C. (2022). Effects of a Low-Carbohydrate, High-Protein Diet on Gut Microbiome Composition in Insulin-Resistant Individuals with Chronic Spinal Cord Injury: Preliminary Results From a Randomized Controlled Trial. Arch. Phys. Med. Rehabil..

[105] Cirnigliaro C.M., La Fountaine M.F., Sauer S.J., Cross G.T., Kirshblum S.C., Bauman W.A. (2024). Preliminary observations on the administration of a glucagon-like peptide-1 receptor agonist on body weight and select carbohydrate endpoints in persons with spinal cord injury: A controlled case series. J. Spinal Cord Med..

[106] Sneij A., Farkas G.J., Carino Mason M.R., Gater D.R. (2022). Nutrition Education to Reduce Metabolic Dysfunction for Spinal Cord Injury: A Module-Based Nutrition Education Guide for Healthcare Providers and Consumers. J. Pers. Med..

[107] Johns J., Krogh K., Rodriguez G.M., Eng J., Haller E., Heinen M., Laredo R., Longo W., Montero-Colon W., Wilson C. (2021). Management of Neurogenic Bowel Dysfunction in Adults after Spinal Cord Injury: Clinical Practice Guideline for Health Care Providers. Top. Spinal Cord Inj. Rehabil..

[108] Calderone A., Cardile D., De Luca R., Quartarone A., Corallo F., Calabrò R.S. (2024). Cognitive, behavioral and psychiatric symptoms in patients with spinal cord injury: A scoping review. Front. Psychiatry.

[109] Moro V., Beccherle M., Scandola M., Aglioti S.M. (2023). Massive body-brain disconnection consequent to spinal cord injuries drives profound changes in higher-order cognitive and emotional functions: A PRISMA scoping review. Neurosci. Biobehav. Rev..

[110] Carlozzi N.E., Graves C.M., Troost J.P., Ehde D.M., Miner J.A., Kratz A.L. (2021). Association of physical and mental symptoms with cognition in people with spinal cord injury. Rehabil. Psychol..

[111] Moro V., Scandola M., Aglioti S.M. (2022). What the study of spinal cord injured patients can tell us about the significance of the body in cognition. Psychon. Bull. Rev..

[112] De Martino M.L., De Bartolo M., Leemhuis E., Pazzaglia M. (2021). Rebuilding Body-Brain Interaction from the Vagal Network in Spinal Cord Injuries. Brain Sci..

[113] Datta N., Hughes A., Modafferi M., Klabunde M. (2025). An FMRI meta-analysis of interoception in eating disorders. NeuroImage.

[114] Dorenkamp M.A., Waldron-Perrine B., Hanks R. (2023). Influence of Acceptance and Commitment Therapy Core Processes on Anxiety and Stress in Persons with Spinal Cord Injury: A Cross-sectional Study. Arch. Phys. Med. Rehabil..

[115] Li M., Lo W.Y., Hu Y., Wang S., Sun T.C., Temesgen W.A., He M., Li Y. (2025). Mindfulness- and acceptance-based interventions for people with spinal cord injury: A scoping review. Spinal Cord.

[116] Bombardier C.H., Azuero C.B., Fann J.R., Kautz D.D., Richards J.S., Sabharwal S. (2021). Management of Mental Health Disorders, Substance Use Disorders, and Suicide in Adults with Spinal Cord Injury: Clinical Practice Guideline for Healthcare Providers. Top. Spinal Cord Inj. Rehabil..

[117] Linardon J., Messer M., Tylka T.L. (2023). Functionality appreciation and its correlates: Systematic review and meta-analysis. Body Image.

[118] Linardon J., Moffitt R., Anderson C., Tylka T.L. (2024). Testing for longitudinal bidirectional associations between self-compassion, self-criticism, and positive body image components. Body Image.

[119] Pastore O.L., Fortier M.S., Maffin J., McBride C.B., Aslam L., Dorvil E., Sweet S.N. (2025). Feasibility, acceptability, and effectiveness of a tailored self-compassion program for peer mentors within Canadian spinal cord injury community service organizations. Disabil. Rehabil..

[120] Armstrong A., Oetinger K., Weimer K., Hönig K. (2024). Digital psychosocial interventions for individuals with spinal cord injury: A scoping review. Front. Psychiatry.

