# Supplementary material for: Body Composition and Body-Image After Spinal Cord Injury: A Scoping Review
Source: Nutrients. 2026 Oct 30;18(21):3604. doi: 10.3390/nu18213604 (PMC13765124; doi:10.3390/nu18213604)
Supplement: Supplementary file 1 [file nutrients-18-03604-s001.zip › nutrients-4628086-supplementary.pdf]

Supplementary Material

Table S1. Review design, protocol, search chronology and responsibilities

Element	Review specification	Interpretive boundary
Purpose and framework	The review maps nutritional status, body composition and eating behaviors alongside distinct body-image constructs after acquired SCI. JBI guidance informed the scoping-review methods [37,40], and PRISMA-ScR guided reporting [39].	The objectives concern concepts, measurements, empirical contributions and gaps, rather than a pooled effect estimate.
Protocol	The protocol was deposited on OSF on 11 September 2026: project XCBHR; DOI 10.17605/OSF.IO/XCBHR.	The review was conceived as a scoping review from the outset. The initial database search followed on 12 September 2026.
Search chronology	The initial search was conducted on 12 September 2026. The update was completed on 16 October 2026 and included APA PsycINFO through APA PsycNet and CINAHL through EBSCOhost.	Final search totals refer to the reconciled update exports. Execution, export and screening are separate steps.
Scope and limits	Acquired traumatic or non-traumatic SCI, age ≥10 years at the eligible assessment, any country or care setting, and original English full-length journal reports.	No lower publication-date limit, minimum sample size, significance threshold or methodological-quality exclusion threshold was applied.
Independent selection	S.C. and A.C. independently assessed every title/abstract and full text before comparing decisions.	Differences were discussed. R.S.C. adjudicated unresolved disagreements. Potentially eligible or insufficiently described records proceeded to further assessment.
Searches, charting and verification	S.C. and A.C. independently checked searches and exports and undertook charting, appraisal and synthesis verification; R.S.C. resolved persistent differences.	Individual study findings were checked against the primary publications. Automated support did not replace the stated researcher responsibilities.
Rayyan	Rayyan supported record management, manual decisions, relevance ordering and automated exclusions subject to researcher assessment.	Affected records remained within title/abstract screening and were counted once. No separate updated numerical category was inferred for automated decisions.
Appraisal	Design- and contribution-specific JBI checklists and property-specific COSMIN standards informed interpretation [40,45–47].	Findings were not excluded through a numerical quality cutoff. Appraisal constrained claims about measurement, confounding, temporal relationships and transferability.
Unit of analysis	The evidence map contains 36 eligible journal reports. Companion reports retain their publication identities, with cohort linkage recorded separately.	Publication counts are not counts of independent cohorts. Shared participants and repeated observations were not pooled as independent persons.
Synthesis	Descriptive mapping and thematic comparison distinguish patient accounts, professional perspectives, direct quantitative relationships and co-assessment.	No meta-analysis, common numerical effect or intervention-effectiveness conclusion was derived.
Contextual sources	Background and Discussion references provide context outside the eligible empirical corpus.	A companion report supplying context is not automatically an additional inclusion. The nutrition-beliefs report by LaVela et al. [87] is contextual because it lacks a distinct body-image contribution in its empirical findings.

Abbreviations: A.C., Andrea Calderone; JBI, Joanna Briggs Institute; OSF, Open Science Framework; PRISMA-ScR, Preferred Reporting Items for Systematic Reviews and Meta-Analyses extension for Scoping Reviews; R.S.C., Rocco Salvatore Calabrò; S.C., Santina Caliri; SCI, spinal cord injury.

Table S2. Population, Concept and Context framework and eligibility boundaries

Domain	Eligible content	Boundary or exclusion
Population	Adolescents and adults aged ≥10 years at assessment with acquired traumatic or non-traumatic SCI.	Congenital disorders and isolated peripheral nerve or nerve-root disorders do not qualify. Age at injury is not substituted for age at assessment.
Concept	An identifiable nutritional domain and a distinct body-image dimension within the empirical contribution.	Both constructs must be present. A comparison group, significant result or direct association test is not mandatory.
Context and perspective	Any setting, country or injury stage. Professional or caregiver accounts may qualify when they explicitly concern nutritional/body-image experiences after SCI.	Professional interpretations, patient accounts and proxy responses remain distinct.
Mixed populations	An acquired-SCI subgroup with separately attributable eligible findings.	A majority SCI diagnosis or adult mean age does not establish eligibility of every participant or pooled theme.
Nutritional domain	Nutritional status or risk, intake, eating practices governing intake, weight or weight change, anthropometry, body composition, or perceived fatness/thinness.	Physical activity, strength, food access, nutrition knowledge or feeding dependence alone are insufficient.
Body-image domain	Appearance evaluation, body satisfaction, weight/shape appraisal, acceptance, appreciation, body esteem, self-presentation or satisfaction with functionality.	General QOL, mood, self-esteem, sexual functioning or sensorimotor ownership alone are insufficient.
Measurement and perception	Measured values, self-reported measurements, recalled change and perceived bodily morphology are charted separately.	Perceived atrophy does not establish measured muscle loss, sarcopenia or malnutrition. Abdominal appearance does not identify its physical cause.
Distinct constructs	A nutritional descriptor and a separate evaluation of the body, even when discussed in the same account.	One weight-dissatisfaction item cannot count simultaneously as the nutritional exposure and body-image outcome.
Quantitative contribution	Distinct nutritional and body-image assessments in the same identifiable SCI sample, with or without a direct relationship test.	Co-assessment indicates that both domains were measured, not that they were associated. Associations with total QOL are not relabeled as body-image associations.
Qualitative contribution	An explicit connection in participant accounts, themes or other empirical findings.	A proposed connection appearing only in an Introduction or Discussion is insufficient. Generic identity or embodiment requires the relevant nutritional and evaluative content.
Companion publications	Each report is eligible on its own empirical contribution. Linked publications may clarify cohort identity, measurement or context.	Complementary information does not make an otherwise ineligible companion an autonomous inclusion or establish a within-person causal sequence.
Publication and language	Original full-length English journal articles, including an official English full-text version.	Reviews, protocols, editorials, conference abstracts, theses, standalone preprints and clinical case reports are excluded. Small qualitative research is classified by its actual design.
Other restrictions	No publication-year, country, sample-size, statistical-significance or quality-score restriction.	English eligibility concerns the report, not the language used during interviews or questionnaire administration.

Age refers to age at the eligible assessment rather than age at injury. Measured body composition, recalled weight change and perceived bodily change remain distinct. A nutritional descriptor and a separate bodily evaluation must be identifiable. Abbreviations: PCC, Population, Concept and Context; QOL, quality of life; SCI, spinal cord injury.

Table S3. Information sources, executable search expressions and search documentation

Information source / procedure	Search expression or procedure	Update records / documentation
PubMed — Advanced Search / query box	("spinal cord injury" OR "spinal cord injuries" OR "spinal cord injured" OR "spinal cord lesion" OR paraplegia OR paraplegic OR tetraplegia OR tetraplegic OR quadriplegia OR quadriplegic) AND ("body image" OR "body satisfaction" OR "body dissatisfaction" OR "body esteem" OR "body appreciation" OR "body acceptance" OR "body perception" OR "appearance satisfaction" OR "appearance concerns" OR "weight concerns" OR "functionality appreciation" OR "self presentation" OR "physical self concept" OR "sexual self esteem" OR "body experience" OR "bodily experience" OR ((nutrition OR nutritional OR diet OR dietary OR eating OR "food avoidance" OR "weight change" OR "weight loss" OR "weight gain" OR "weight management" OR "body composition" OR "body fat" OR "lean mass" OR "muscle mass" OR obesity OR malnutrition) AND ("quality of life" OR psychosocial OR "self esteem" OR "self concept" OR qualitative OR interview OR interviews)))	Search update completed 16 October 2026. 514 records.
Cochrane Library — query box / Search Manager	("spinal cord injury" OR "spinal cord injuries" OR "spinal cord injured" OR "spinal cord lesion" OR paraplegia OR paraplegic OR tetraplegia OR tetraplegic OR quadriplegia OR quadriplegic) AND ("body image" OR "body satisfaction" OR "body dissatisfaction" OR "body esteem" OR "body appreciation" OR "body acceptance" OR "body perception" OR "appearance satisfaction" OR "appearance concerns" OR "weight concerns" OR "functionality appreciation" OR "self presentation" OR "physical self concept" OR "sexual self esteem" OR "body experience" OR "bodily experience" OR ((nutrition OR nutritional OR diet OR dietary OR eating OR "food avoidance" OR "weight change" OR "weight loss" OR "weight gain" OR "weight management" OR "body composition" OR "body fat" OR "lean mass" OR "muscle mass" OR obesity OR malnutrition) AND ("quality of life" OR psychosocial OR "self esteem" OR "self concept" OR qualitative OR interview OR interviews)))	Search update completed 16 October 2026. 203 records: 95 CENTRAL trials, 98 Cochrane Reviews, 9 protocols and 1 Clinical Answer.
Web of Science Core Collection — Topic (TS)	TS=(("spinal cord injury" OR "spinal cord injuries" OR "spinal cord injured" OR "spinal cord lesion" OR paraplegia OR paraplegic OR tetraplegia OR tetraplegic OR quadriplegia OR quadriplegic) AND ("body image" OR "body satisfaction" OR "body dissatisfaction" OR "body esteem" OR "body appreciation" OR "body acceptance" OR "body perception" OR "appearance satisfaction" OR "appearance concerns" OR "weight concerns" OR "functionality appreciation" OR "self presentation" OR "physical self concept" OR "sexual self esteem" OR "body experience" OR "bodily experience" OR ((nutrition OR nutritional OR diet OR dietary OR eating OR "food avoidance" OR "weight change" OR "weight loss" OR "weight gain" OR "weight management" OR "body composition" OR "body fat" OR "lean mass" OR "muscle mass" OR obesity OR malnutrition) AND ("quality of life" OR psychosocial OR "self esteem" OR "self concept" OR qualitative OR interview OR interviews))))	Search update completed 16 October 2026. 470 records.
APA PsycINFO — APA PsycNet, Any Field	(Any Field: "spinal cord injury" OR Any Field: "spinal cord injuries" OR Any Field: "spinal cord injured" OR Any Field: "spinal cord lesion" OR Any Field: paraplegia OR Any Field: paraplegic OR Any Field: tetraplegia OR Any Field: tetraplegic OR Any Field: quadriplegia OR Any Field: quadriplegic) AND (Any Field: "body image" OR Any Field: "body satisfaction" OR Any Field: "body dissatisfaction" OR Any Field: "body esteem" OR Any Field: "body appreciation" OR Any Field: "body acceptance" OR Any Field: "body perception" OR Any Field: "appearance satisfaction" OR Any Field: "appearance concerns" OR Any Field: "weight concerns" OR Any Field: "functionality appreciation" OR Any Field: "self presentation" OR Any Field: "physical self concept" OR Any Field: "sexual self esteem" OR Any Field: "body experience" OR Any Field: "bodily experience" OR ((Any Field: nutrition OR Any Field: nutritional OR Any Field: diet OR Any Field: dietary OR Any Field: eating OR Any Field: "food avoidance" OR Any Field: "weight change" OR Any Field: "weight loss" OR Any Field: "weight gain" OR Any Field: "weight management" OR Any Field: "body composition" OR Any Field: "body fat" OR Any Field: "lean mass" OR Any Field: "muscle mass" OR Any Field: obesity OR Any Field: malnutrition) AND (Any Field: "quality of life" OR Any Field: psychosocial OR Any Field: "self esteem" OR Any Field: "self concept" OR Any Field: qualitative OR Any Field: interview OR Any Field: interviews)))	Search update completed 16 October 2026. 15 records.

Information source / procedure	Search expression or procedure	Update records / documentation
Embase.com — query builder, All fields	('spinal cord injury' OR 'spinal cord injuries' OR 'spinal cord injured' OR 'spinal cord lesion' OR 'paraplegia' OR 'paraplegic' OR 'tetraplegia' OR 'tetraplegic' OR 'quadriplegia' OR 'quadriplegic') AND ('body image' OR 'body satisfaction' OR 'body dissatisfaction' OR 'body esteem' OR 'body appreciation' OR 'body acceptance' OR 'body perception' OR 'appearance satisfaction' OR 'appearance concerns' OR 'weight concerns' OR 'functionality appreciation' OR 'self presentation' OR 'physical self concept' OR 'sexual self esteem' OR 'body experience' OR 'bodily experience' OR (('nutrition' OR 'nutritional' OR 'diet' OR 'dietary' OR 'eating' OR 'food avoidance' OR 'weight change' OR 'weight loss' OR 'weight gain' OR 'weight management' OR 'body composition' OR 'body fat' OR 'lean mass' OR 'muscle mass' OR 'obesity' OR 'malnutrition') AND ('quality of life' OR 'psychosocial' OR 'self esteem' OR 'self concept' OR 'qualitative' OR 'interview' OR 'interviews')))	Search update completed 16 October 2026. 1,109 records exported from the Articles tab. All fields searched the entered terms as free text, without Emtree mapping or explosion. No database search limits or filters were applied. The Clinical Trials export is listed separately.
CINAHL — EBSCOhost, all fields	(("spinal cord injury" OR "spinal cord injuries" OR "spinal cord injured" OR "spinal cord lesion" OR paraplegia OR paraplegic OR tetraplegia OR tetraplegic OR quadriplegia OR quadriplegic)) AND (("body image" OR "body satisfaction" OR "body dissatisfaction" OR "body esteem" OR "body appreciation" OR "body acceptance" OR "body perception" OR "appearance satisfaction" OR "appearance concerns" OR "weight concerns" OR "functionality appreciation" OR "self presentation" OR "physical self concept" OR "sexual self esteem" OR "body experience" OR "bodily experience" OR ((nutrition OR nutritional OR diet OR dietary OR eating OR "food avoidance" OR "weight change" OR "weight loss" OR "weight gain" OR "weight management" OR "body composition" OR "body fat" OR "lean mass" OR "muscle mass" OR obesity OR malnutrition)) AND (("quality of life" OR psychosocial OR "self esteem" OR "self concept" OR qualitative OR interview OR interviews))))	Search update completed 16 October 2026. 311 records retrieved through EBSCOhost. Apply equivalent subjects was disabled. No database search limits or filters were applied.
Scopus — query builder, All fields (ALL)	("spinal cord injury" OR "spinal cord injuries" OR "spinal cord injured" OR "spinal cord lesion" OR paraplegia OR paraplegic OR tetraplegia OR tetraplegic OR quadriplegia OR quadriplegic) AND ("body image" OR "body satisfaction" OR "body dissatisfaction" OR "body esteem" OR "body appreciation" OR "body acceptance" OR "body perception" OR "appearance satisfaction" OR "appearance concerns" OR "weight concerns" OR "functionality appreciation" OR "self presentation" OR "physical self concept" OR "sexual self esteem" OR "body experience" OR "bodily experience" OR ((nutrition OR nutritional OR diet OR dietary OR eating OR "food avoidance" OR "weight change" OR "weight loss" OR "weight gain" OR "weight management" OR "body composition" OR "body fat" OR "lean mass" OR "muscle mass" OR obesity OR malnutrition) AND ("quality of life" OR psychosocial OR "self esteem" OR "self concept" OR qualitative OR interview OR interviews)))	Search update completed 16 October 2026. 23,993 records.
Embase trial-registration export	The same Embase search supplied the Articles and Clinical Trials tabs. Results from the two tabs were exported separately without an additional registration-specific query or any additional filters or restrictions.	282 trial-registration records exported from the Clinical Trials tab, kept separate from the 1,109 records exported from the Articles tab. ClinicalTrials.gov was not searched directly.
Conceptual structure	SCI AND [body image OR (nutrition AND psychosocial/experiential terms)]. Synonyms within blocks are combined with OR.	The body-image branch can retrieve nutritional content reported only in the full text. The nutritional branch does not require explicit body-image indexing. Eligibility still requires both empirical domains.
Field selection and term mapping	APA PsycINFO used Any Field through APA PsycNet. Scopus used All fields (ALL). Embase.com used the query builder with All fields, searching free text without Emtree mapping or explosion. CINAHL used all fields through EBSCOhost, with Apply equivalent subjects disabled. Web of Science used Topic (TS).	No database search limits or filters were applied. Other interface options were not manually modified. Eligibility criteria, including the English-language requirement, were assessed during study selection.
Coverage and language	Searches extended from inception to the final search date. English full-text eligibility was assessed during selection.	Article-language eligibility is distinct from search-interface filters. No undocumented date, age or language filter was attributed to a database.

Information source / procedure	Search expression or procedure	Update records / documentation
Scopus export reconciliation	Two export segments contained 16,074 and 16,321 rows. Repeated Scopus identifiers identified 8,402 rows present in both segments.	16,074 + 16,321 − 8,402 = 23,993 source records. Segment repeats were removed before the bibliographic-deduplication stage.
Source totals	The reconciled update contained 26,615 database records and 282 registration-export records.	26,897 identified records. The source components above sum to this total.
Known-report coverage check	Known pertinent reports were compared with the retrieved records using bibliographic identifiers and titles.	Two eligible reports not represented in the database exports were retained through reference checking: Astorino and Harness [51] and Thrussell et al. [66]. This is a coverage check, not a validated sensitivity estimate.
Reference and companion checking	References and explicit publication links were examined to identify pertinent reports and clarify shared cohorts.	The two additional eligible reports were counted in the other-sources branch. Matching authors or institutions alone did not establish participant overlap.
Independent search checks	S.C. and A.C. checked the strategies and exports independently, considering concept translation, Boolean logic, terms, fields and limits [42].	R.S.C. adjudicated unresolved differences. These checks are not described as a completed formal PRESS assessment without a documented checklist.
Chronology	The protocol was deposited on 11 September 2026, the initial search ran on 12 September 2026 and the update was completed on 16 October 2026.	The review remained a scoping review throughout. Final selection counts refer to the updated search rather than the initial export alone.
Coverage qualification	PsycINFO broadens psychological coverage, while CINAHL, searched through EBSCOhost, extends nursing and allied-health coverage.	The selected databases provide complementary coverage of clinical, psychological, nursing and allied-health research.

The protocol was deposited on OSF on 11 September 2026. The initial search was conducted on 12 September 2026 and the search update was completed on 16 October 2026. Expressions reproduce the database-specific query-builder outputs. TS denotes Topic in Web of Science. Any Field is the field used for APA PsycINFO through APA PsycNet. ALL denotes All fields in Scopus. Embase.com used the query builder with All fields. CINAHL was searched through EBSCOhost across all fields, with Apply equivalent subjects disabled. The Cochrane export contained trials, reviews, protocols and a Clinical Answer, with eligibility determined after retrieval. Abbreviations: APA, American Psychological Association; CENTRAL, Cochrane Central Register of Controlled Trials; SCI, spinal cord injury.

Table S4. Selection procedures and reconciled PRISMA counts

Stage / procedure	Decision or count	Number	Counting or interpretation rule
Duplicate assessment	DOI, PMID and bibliographic details, with manual review of uncertain matches [43].	Not applicable	Duplicate records of one publication were removed. Companion reports remained bibliographically distinct.
Title/abstract assessment	Every title and abstract was independently assessed by S.C. and A.C. before comparison.	24,642	Potentially eligible or insufficiently described records proceeded to further assessment. An absent abstract was not itself an exclusion reason.
Rayyan support	Management, manual decisions, relevance ordering and automatic exclusions with researcher verification.	Within the screening total	Affected records were assessed within the same stage and counted once. The update did not provide a separate verified automated-exclusion subtotal.

Stage / procedure	Decision or count	Number	Counting or interpretation rule
Full-text assessment	Every full text was independently assessed by S.C. and A.C.; persistent disagreements were adjudicated by R.S.C.	591 across both branches	One primary exclusion reason was retained per report. Additional concerns did not increase the count.
Publication type	Original full-length journal research; design-specific classification of small samples.	Decision rule	Clinical case reports are distinct from small qualitative or cross-sectional studies.
Language	English full text or an official English version.	Decision rule	Interview language does not determine article-language eligibility.
Population	Acquired SCI and eligible age at assessment; separately attributable SCI findings in mixed samples.	Decision rule	Neither mean age nor a majority SCI diagnosis establishes eligibility of pooled findings.
Nutritional concept	Nutritional status/risk, intake, eating practices, weight, anthropometry or composition.	Decision rule	Physical activity, strength or generic feeding dependence alone does not satisfy the criterion.
Body-image concept	A distinct instrument, subscale, explicit item or qualitative dimension.	Decision rule	General QOL, self-esteem, sexual function or ownership alone does not qualify.
Empirical contribution	Separate co-assessed constructs in quantitative work; an explicit empirical connection in qualitative work.	Decision rule	Background claims and unlinked samples do not establish an eligible contribution.
Identification: databases	Records in bibliographic-database exports.	26,615	PubMed 514; Web of Science 470; Embase journal records 1,109; Scopus 23,993; APA PsycINFO 15; CINAHL (EBSCOhost) 311; Cochrane Library 203.
Identification: registrations	Separate trial-registration export.	282	Obtained through Embase and kept distinct from journal records.
Identification: total	Reconciled source records.	26,897	26,615 + 282. Scopus segment repeats had already been removed.
Before screening	Bibliographic duplicates removed.	2,255	26,897 − 2,255 = 24,642 records for screening.
Title/abstract exclusion	Ineligible publication type.	12,562	One primary reason per excluded record.
Title/abstract exclusion	Ineligible population.	8,651	One primary reason per excluded record.
Title/abstract exclusion	No distinct body-image domain.	1,731	One primary reason per excluded record.
Title/abstract exclusion	No eligible nutritional domain.	1,040	One primary reason per excluded record.
Title/abstract exclusion	Outside nutrition/body-image scope.	50	Historical general scope category, retained separately from the two specific domain categories.
Title/abstract exclusion	Ineligible language.	15	One primary reason per excluded record.
Title/abstract exclusion	Required qualitative connection absent.	4	One primary reason per excluded record.
Title/abstract exclusions: total	Records excluded.	24,053	12,562 + 8,651 + 1,731 + 1,040 + 50 + 15 + 4.
Retrieval: database branch	Reports sought for retrieval.	589	24,642 − 24,053.
Retrieval: database branch	Reports not retrieved at completion.	0	Final retrieval status after completion of eligibility assessment.

Stage / procedure	Decision or count	Number	Counting or interpretation rule
Eligibility: database branch	Reports assessed.	589	Reports sought minus reports not retrieved.
Full-text exclusion	Ineligible publication type.	21	One primary reason per excluded report.
Full-text exclusion	Ineligible language.	17	One primary reason per excluded report.
Full-text exclusion	Ineligible population.	71	One primary reason per excluded report.
Full-text exclusion	No eligible nutritional domain.	109	One primary reason per excluded report.
Full-text exclusion	No distinct body-image domain.	118	One primary reason per excluded report.
Full-text exclusion	Required empirical connection absent.	12	One primary reason per excluded report.
Full-text exclusion	Outside the review scope.	207	Reports did not meet the specified combined scope of the review.
Full-text exclusions: database total	Reports excluded.	555	21 + 17 + 71 + 109 + 118 + 12 + 207.
Included: database branch	Eligible reports.	34	589 − 555.
Other sources	Reports identified through reference checking.	2	Both were retrieved, assessed and eligible; none were excluded.
Eligibility: combined branches	Reports assessed.	591	589 database/registration-export reports + 2 other-source reports.
Included: total	Eligible journal reports.	36	34 database/registration-export reports + 2 other-source reports; 25 retained original reports + 11 additions.
Unit of analysis	Publications, not independent populations.	36 reports	Cohort overlap is recorded separately. No pooled number of unique participants or independent cohorts is asserted.

Each exclusion count represents one primary reason per record or report at the relevant stage. Exclusion categories are defined separately at the record and report stages. The full-text scope category contains 207 reports that did not meet the specified review scope. Reports obtained through reference checking are shown separately from the database and registration-export branch. Abbreviations: A.C., Andrea Calderone; DOI, digital object identifier; PMID, PubMed identifier; PRISMA, Preferred Reporting Items for Systematic Reviews and Meta-Analyses; R.S.C., Rocco Salvatore Calabrò; S.C., Santina Caliri; SCI, spinal cord injury.

Table S5. Data charting and evidence synthesis procedures

Field / procedure	Information charted	Application to the synthesis
Bibliographic identity	Authors, journal-issue and online-first years, title, journal, DOI/PMID and related reports.	One publication retains one identity. Online-first and issue years are not counted as separate reports.
Design and setting	Study question, design, recruitment, country of study conduct and care setting.	The contribution used in the review determines classification, including the relevant component of mixed-methods work.
Participants and denominators	Recruited, analyzed, clinical and interview samples; eligible subgroups; age, sex/gender, injury features and time since SCI.	Patients, professionals and proxies remain separate. Repeated visits, observations and interviews are not additional participants.
Nutritional constructs	Status or risk, intake, eating practices, weight, anthropometry and body composition, with methods, units and timing.	Measured, self-reported, recalled and perceived information is identified explicitly.
Body-image constructs	Appearance, weight/shape appraisal, satisfaction, acceptance, esteem, self-presentation or functionality satisfaction; instrument, subscale, item or theme.	General QOL or mood is not substituted for a body-image outcome. One item is not used twice to establish both domains.
Contribution classification	Patient qualitative connection, professional qualitative connection, direct quantitative relationship or quantitative co-assessment.	The final corpus contains 24 patient qualitative, 3 professional qualitative and 9 quantitative reports. Co-assessment is not an association finding.
Quantitative results	Relevant coefficient, direction, uncertainty, analytical denominator, covariates and assessment time when reported.	The three relational reports are described separately from six co-assessment reports. Unresolved source inconsistencies are retained with an explanatory note.
Qualitative results	The nutritional/body-image connection, participant perspective, theme and supporting account.	Participant statements are distinguished from author interpretation. Theme frequency is not population prevalence.
Measurement support	Scoring, administration, language, adaptation and relevant validity/reliability evidence.	Whole-scale consistency is not transferred to an individual domain. Bilingual administration does not establish measurement invariance.
Independent charting	S.C. and A.C. independently charted and checked the primary reports before comparing entries.	Discrepancies were discussed; R.S.C. adjudicated unresolved differences. Unreported information was not imputed.
Descriptive mapping	Publication period, geography, design, population and assessment approach.	Denominators are explicit and categories may overlap only when identified as non-exclusive. Shared samples are not summed.
Thematic comparison	Shared, contrasting and contradictory meanings across empirical contributions [44,48].	Themes concern reported experiences and their context, not a pooled numerical effect or a proposed mechanism validated by the review.
Perspective separation	Patient, professional, caregiver and proxy findings.	Professional concerns about disordered eating or body dysmorphia are not verified patient diagnoses or prevalence estimates.
Cohort linkage	Explicit recruitment links, matching participant identifiers, sample characteristics and assessment periods.	Confirmed and possible overlap are distinguished. Shared authors or location alone are insufficient.
Subgroup attribution	SCI diagnosis, age at assessment, sex/gender, injury stage and identifiable accounts within mixed samples.	Pooled mixed-disability findings and full-cohort measurements are not automatically assigned to the eligible subgroup.
Appraisal	Tool/version, relevant contribution or result, item/property judgments and rationale [40,45–47].	Reflexivity and context inform qualitative transferability; measurement, confounding, missingness and timing constrain quantitative interpretation. No cross-design score is used.
Missingness and reporting conflicts	Unavailable details, inconsistent denominators and conflicting statistics.	The reported value is preserved when unresolved. A plausible explanation is not presented as a verified correction.
Rehabilitation applications	Assessment topics and research priorities developed from the empirical map and relevant contextual sources.	The framework is conceptual and untested. Its acceptability, feasibility and effects require evaluation.

Nutritional and body-image constructs were charted separately, then classified by the empirical contribution available in each report. Missing information was retained as not reported, and participant overlap was not resolved by assuming independence. Rehabilitation applications are conceptual interpretations of the evidence map and were not evaluated as interventions. Abbreviations: A.C., Andrea Calderone; DOI, digital object identifier; NR, not reported; PMID, PubMed identifier; R.S.C., Rocco Salvatore Calabrò; S.C., Santina Caliri; SCI, spinal cord injury.

Table S6. Detailed report findings, analytical denominators and contribution-specific limitations.

Report and contribution	Nutritional or eating domain	Body-image domain	Source-supported findings and denominators	Interpretive qualification
Astorino and Harness, 2020 [51]; Longitudinal change and exploratory association	DXA composition, waist and four-day dietary records	BSS and appearance component	Body satisfaction and appearance satisfaction improved over six months while body fat percentage increased and whole-body fat-free mass did not clearly change. An exploratory body-fat association with change in satisfaction was reported but its denominator and p value are uncertain.	25 completers; no comparator. Correlation denominator/timing and reported r/p correspondence remain uncertain.
Abedi et al., 2023 [52]; Quantitative co-assessment	No independent anthropometric or nutritional diagnosis; distinct IBS-QOL food-avoidance items retained	Separate body-image items retained; feeling-fat item removed	Food-avoidance and body-image items were measured in the same SCI sample, but their direct association was not tested. Item 5 on feeling fat was removed before construct validation after patient/professional feedback. Overall internal consistency was α=0.9684. Exploratory analysis yielded six factors, whereas the original BI items split across factors. CFA fit remained poor despite improving relative to the original model (alternative CFI 0.6149, RMSEA 0.1462).	106 patients; total alpha is not BI-domain validation, and structural fit remained limited
Rosito et al., 2002 [53]; Quantitative co-assessment	Recalled weightloss, diet and eating enjoyment	Study-specific body-image domain	Nutritional/weight items and a distinct body-image domain were co-assessed. Recalled weight-loss frequency was lower after stoma, while authors reported no statistically significant body-image-domain change. No direct weight–image association was tested.	Co-assessment without direct NI–BI test; retrospective recall and limited instrument validation.
LaVela et al., 2021 [54]; Patient qualitative connection	Weight-management targets and recalled weight	Appearance motivations and clothing fit	Appearance, health and mobility motivated weight management. Participants valued feedback on progress and desired reductions in specific body regions. Goals represent preferences rather than achieved weight loss or improved body image.	Goals and preferences, not achieved changes. Shares interviews with companion communication report.
LaVela et al., 2024 [55]; Patient qualitative connection	Perceived excess weight and recalled gain	Appearance concerns, clothing fit and mirror appraisal	Perceived excess weight was linked to dissatisfaction with appearance and clothing fit, impaired intimacy and negative self-appraisal alongside movement and health concerns.	Qualitative sample. The reported 42% is a coded theme frequency, not prevalence.
LaVela et al., 2024 [56]; Professional qualitative connection	Provider concerns about appetite suppression and eating adequacy	Perceived body-image vulnerability	Providers expressed concerns that appetite suppression and reliance on medication could undermine eating adequacy or interact with body-image vulnerability. These are perceptions of potential barriers, not observed medication harms or eating-disorder incidence.	Provider perceptions only. No evidence here of drug-induced body-image harm or eating-disorder prevalence.
Kehn and Kroll, 2009 [57]; Patient qualitative connection	Recalled weightgain, loss and regain	Positive/negative appraisal of weight changes	Weight change and its emotional meaning shaped exercise motivations. A woman described distress with gained weight, while a man contrasted feeling good after weightloss with disappointment after regain.	Subjective narratives within an exercise study; no quantified composition or direct association test.
Bailey et al., 2015 [58]; Patient qualitative connection	Eating as self-care, weight and bowel-management routines	Body acceptance, appreciation and gratitude	Eating as self-care and coordination of diet, bowel routines and exercise were described as part of accepting and appreciating the body. The proposed relationships are qualitative and preliminary.	Qualitative preliminary model; same nine-person cohort as Bailey 2016.

Report and contribution	Nutritional or eating domain	Body-image domain	Source-supported findings and denominators	Interpretive qualification
Bailey et al., 2016 [59]; Patient qualitative connection	Self-reported weight and perceived morphological change	Appearance, stigma and self-presentation	Perceived weight gain, abdominal change and muscularity were linked to appearance concerns, clothing choices and attempts to manage stigma. Weight concerns also involved mobility and accessibility.	Subjective accounts; shares nine-person cohort with Bailey 2015.
Bailey et al., 2018 [60]; Patient qualitative connection	Recalled weight loss; weight explicitly not measured Diet-adherence experiences, support, autonomy and access barriers	Appearance benefits and improved feelings about oneself in a focal account	A woman linked approximately 35 pounds of recalled weight loss to appearance benefits and feeling better about herself. Other participants emphasized functional benefits, family/peer support, autonomy, costs and reduced motivation once formal study support ended.	6 unique participants; selected qualitative follow-up does not estimate dietary efficacy
Kathnelson et al., 2020 [61]; Patient qualitative connection	Perceived muscle loss, thinner limbs and abdominal enlargement No quantified intake	Appearance distress and concerns about attractiveness in intimate relationships	Men connected perceived abdominal enlargement and thinning limbs with difficulty accepting appearance and fears about attractiveness. Descriptions of body change were distinct from evaluations of the body and anticipated rejection. Broader sexuality narratives emphasized intimacy and emotional connection.	6 men; perceived muscular size is not measured muscle mass or diagnosed sarcopenia
Chau et al., 2008 [62]; Patient qualitative connection	Perceived muscle/leg-shape and abdominal changes No quantified intake	Distress about clothing fit and mirror appearance, with evolving acceptance	A participant connected perceived muscle atrophy and abdominal/leg-shape changes with clothing no longer fitting as before, distress at mirror appearance and reluctance to go outside. The framework described movement between discomfort and comfort, shaped by social interaction and self-representation.	15 women from a shared parent cohort; perceived atrophy is not measured muscle loss
Sheldon et al., 2011 [63]; Patient qualitative connection	Perceived thinness in a narrative	Appearance, clothing and self-concept	Men described adapting appearance and clothing to changed bodies and public judgments. A man explicitly linked perceived thinness to concern about exposed ankles and trouser fit.	Perceived morphology only; secondary analysis and shared parent cohort.
Renwick et al., 2018 [64]; Patient qualitative connection	Perceived overweight in a focal participant account No quantified intake	Feeling better about appearance and managing public staring	A participant linked not being overweight and feeling good about appearance to greater comfort with public staring. The wider analysis described heterogeneous responses to staring, including avoidance, confrontation and positive self-presentation.	30 men from an overlapping parent cohort; focal account is not a prevalence estimate
Barrett et al., 2023 [65]; Patient qualitative connection	One participant recalled gain, abdominal enlargement and subsequent weight loss No quantified intake	Clothed-body confidence improved after weight loss, while unclothed discomfort persisted	One woman described weight gain and a para-belly, followed by weight loss that improved her feelings when clothed but not when unclothed. This occurred within a theme on diminished sexual confidence, alongside wider experiences of stigma, communication and partner relationships.	20 participants; the focal account is not a measured trajectory or population frequency
Thrussell et al., 2018 [66]; Patient qualitative connection	Recalled weight/shape changes; food and fluid avoidance	Body acceptance and sexual self-image	Women linked recalled weight and shape changes to difficulty accepting their appearance, mirror/photo avoidance and altered sexual confidence. Food/fluid avoidance also appeared as continence-management practices, which should not automatically be attributed to appearance concerns.	Qualitative accounts; eating avoidance for continence and appearance concerns are distinct motives.
Martinsen et al., 2008 [67]; Patient qualitative connection	Assisted eating, food avoidance and meal devices	Embodied self-image and meal-related presentation	Food choices and assistive devices were negotiated to preserve self-recognition and avoid humiliating or stigmatizing presentation during assisted meals.	No quantified intake or nutrition–image association;16 persons, despite repeated interviews.

Report and contribution	Nutritional or eating domain	Body-image domain	Source-supported findings and denominators	Interpretive qualification
Buchtler et al., 2021 [68]; Patient qualitative connection	Subjectively smaller muscles	Body experience, appearance and self-recognition	A participant described smaller muscles while retaining a recognizable and continuous self-image. Broader themes addressed autonomy, body-related emotions and communication.	Individual qualitative narrative; no composition measurement or nutritional diagnosis.
Desneves et al., 2026 [69]; Patient qualitative connection	BIS/anthropometry in 21 clinical patients; interview accounts of weight and muscle changes; dietary quantity and protein choices discussed after composition feedback	Unwanted weight/shape concerns and encouraging interpretation of muscle gain	The interview theme on self-image and lifestyle distinguished perceived weight/muscle changes from worries about unwanted weight and abdominal shape. Patients described adjusting food quantity and protein choices after composition feedback, and one described gain interpreted as muscle as encouraging. Clinicians discussed utility and difficulty of providing feedback. Overall pathway adherence was incomplete, with all scheduled BIS measurements achieved in 43% of patients.	12 patient interviews; clinical measurements are contextual, without a composition–BI association test
Beckwith and Yau, 2013 [70]; Patient qualitative connection	Perceived muscle loss, excess weight and abdominal change No quantified intake	Appearance distress, femininity and evolving body acceptance	Women described muscle loss, excess weight and abdominal appearance among body-image barriers to sexual identity. A participant distinguished the loss of muscular contours from her negative appearance judgment. Experiences also included growing body acceptance and sexual recovery rather than a uniform negative course.	7 women; perceived fat/muscle changes are not objective composition outcomes
Parker and Yau, 2012 [71]; Patient qualitative connection	Perceived abdominal enlargement and absent musculature No quantified intake	Dislike of stomach and changed body/sexual self-image	A participant explicitly linked a fat-appearing abdomen and perceived lack of muscle with dislike of that part of her body. Other appearance concerns, anticipated social judgments and self-presentation were embedded in reconstruction of sexuality.	4 women; abdominal appearance is not measured adiposity
Kim et al., 2013 [72]; Quantitative co-assessment	Measured waist classification; AO perception subgroup 84 45/84 (53.6%) reported dietary modification for weight control	42/84 (50.0%) perceived themselves as fat/very fat	Among 84 participants classified as abdominally obese, 32 (38.1%) perceived themselves as fat and 10 (11.9%) as very fat, together 42/84 (50.0%). The corresponding general-population AO group had 1,562/2,036 (76.7%) in these categories; the body-size response distribution differed ($p<0.001$). Of 84 SCI AO participants, 45 (53.6%) reported dietary modification for weight control. These are descriptive classifications and comparisons rather than adjusted NI–BI effect estimates.	371 enrolled; analysis denominators inconsistent. Perceived size is not body dissatisfaction
Li and Yau, 2006 [73]; Patient qualitative connection	Perceived leg thinning and abdominal/chin size in SCI-attributable accounts No quantified intake	Appearance embarrassment, unattractiveness and concealment	Identifiable women with acquired SCI described embarrassment at thin legs and appearance-related concealment. A woman with paraplegia linked perceived abdominal/chin fatness to feeling unattractive. These findings can be extracted separately from the four poliomyelitis narratives.	Only 6 SCI women are eligible; whole-sample frequencies are not SCI-specific
Potgieter and Khan, 2005 [74]; Patient qualitative connection	Recalled weight loss and perceived thinness; no anthropometry Dieting described as a personal self-care practice	Attractiveness concerns, body acceptance and sexual self-esteem	An adolescent described recalled weight loss and thinness together with concern about attractiveness and how others viewed the body. Other accounts connected dieting and body-care practices with acceptance, demonstrating varied rather than uniformly negative experiences.	7 participants; no nutritional diagnosis, effect estimate or prevalence inference
Duarte Torres et al., 2016 [75]; Patient qualitative connection	Retrospectively reported major weight loss and changed appearance Hospital-food dissatisfaction/rejection described; no quantified intake	Transformed self-image and psychological distress	A participant linked a recalled major weight reduction and skeletal appearance to transformation of self-image and psychological distress. Broader themes concerned relationships, the burden of disability and adaptation to new bodily conditions.	12 participants; only 2 adolescents; recalled weight is not a verified trajectory

Report and contribution	Nutritional or eating domain	Body-image domain	Source-supported findings and denominators	Interpretive qualification
Bassett and Martin Ginis, 2009 (men) [76]; Direct quantitative association	Measured weight, supine waist circumference and height, with BIA-derived body-fat percentage.	Adult Body Satisfaction Questionnaire appearance and functional satisfaction subscales, plus a separate single item for perceived body-image impact on quality of life.	Canada. Fifty men from SHAPE-SCI, mean age 42.3 years and at least 12 months after SCI, underwent measured weight, supine waist circumference and BIA-derived fat assessment alongside separate appearance and functional satisfaction subscales. Appearance correlations were r=0.08 for weight, 0.21 for waist and 0.00 for fat percentage. Functional correlations were r=0.22, 0.21 and 0.08, respectively. All were reported nonsignificant. Weight and fat assessments used n=50, whereas waist circumference used n=49. No confidence intervals or exact NI–BI p values were provided. The principal activity/QoL model is not a nutritional effect estimate. This report shares the SHAPE-SCI parent study with the separate women-only pilot.	Unadjusted cross-sectional correlations do not establish causal direction. Nonsignificant estimates without confidence intervals do not demonstrate absence of a relationship.
Bassett et al., 2009 (women) [77]; Direct quantitative association	Measured body weight using a wheelchair scale, supine waist circumference at the lowest rib, and BIA-derived body-fat percentage.	Adult Body Satisfaction Questionnaire appearance and functional satisfaction subscales, scored separately on a seven-point scale from−3 to+3.	Canada. This cross-sectional SHAPE-SCI pilot included 11 women, mean age 39.3 years, at least 12 months after SCI. Body weight, supine waist circumference and BIA-derived body-fat percentage were measured separately from Adult Body Satisfaction Questionnaire appearance and functional satisfaction. One-tailed Spearman correlations linked appearance satisfaction inversely with waist circumference,ρ=−0.64, p<0.05. The body-fat correlation,ρ=−0.46, met only the exploratory p<0.10 threshold; weight,ρ=−0.33, was not significant. Functional correlations were not significant. The very small sample, multiple exploratory correlations and absence of adjusted models limit interpretation. The journal heading CASE REPORT does not describe a single clinical case.	Small cross-sectional substudy with unadjusted exploratory correlations. One-tailed tests and the p<0.10 exploratory threshold must be reported. The CASE REPORT section heading does not change the documented N=11 cross-sectional design into a single clinical case report.
Coggrave et al., 2012 [78]; Quantitative co-assessment	Self-reported dietary manipulation to assist bowel care before and after stoma formation. This was not a dietary-intake or body-composition assessment.	Four study-specific questions concerning the stoma, appearance, clothing and relationships, plus the Tennessee Self-Concept Scale physical-self subscale.	United Kingdom. A retrospective postal survey across five SCI centres received 104 responses from 149 invitees, with 92 respondents analysed after 12 were excluded. Participants were 64 men and 28 women aged 24–86 years. Dietary manipulation for bowel care was assessed separately from stoma/appearance questions and Tennessee Self-Concept Scale physical-self ratings. Reported dietary manipulation decreased after stoma formation, p=0.001, and 33% reported appearance changes. No direct diet–body-image relationship was tested. Recall bias, self-selection and variable outcome denominators limit interpretation. The published 83(70%) stoma-attitude statement is internally inconsistent, and the TSCS summary is labelled differently in the table and Discussion.	Retrospective recall, self-selection and varying denominators constrain interpretation. Dietary-management changes are not a controlled intervention effect. Published body-image count/percentage and TSCS summary-statistic labels are inconsistent.

Report and contribution	Nutritional or eating domain	Body-image domain	Source-supported findings and denominators	Interpretive qualification
LaVela et al., 2021 (healthy eating) [79]; Patient qualitative belief	Personal meanings of healthy eating, including food content, portions, mindful eating and eating for health.	A narrow aspiration for better skin appearance in one participant account. No formal body-image satisfaction/acceptance instrument or global assessment.	United States. Twenty-five community-dwelling adults with SCI, including 24 men, aged 26–83 years participated in thematic interviews about healthy eating and physical activity. Nutritional content included food choices, portions, mindfulness and eating to enhance health. The body-image contribution was peripheral: one participant with C6-complete SCI described healthy eating partly in terms of desired improvements in skin appearance. This was an appearance-related belief within a personal-meaning account, not a global body-image assessment, measured skin benefit or demonstrated nutritional effect. The late 2018 patient sample may overlap companion weight-management publications from the same project.	The appearance contribution is peripheral and consists of a food–skin-appearance belief. No measured skin-health or body-image improvement was demonstrated. The sample is 96% male and may overlap companion patient analyses from the same project.
LaVela et al., 2025 [80]; Professional qualitative link	Professional accounts of diet quality, calorie and protein needs, unintended weight gain, fat accumulation and muscle loss. No patient nutritional or body-composition measurements were collected.	Provider-described body-image concerns associated with altered body habitus. Possible body dysmorphia was discussed, without a diagnostic assessment or patient scale.	United States. Twelve healthcare providers from 11 Veterans Health Administration hospitals participated in descriptive qualitative interviews in May–July 2023. The sample comprised nine men and three women, primarily dietitians. Providers connected poor diet, perceived fat accumulation and muscle loss with body-image concerns. Body image also appeared within perceived diet–mental-health relationships, whose causal direction providers explicitly questioned. No patients were sampled, and no nutritional or body-image outcomes were measured. The report contributes professional interpretations rather than patient prevalence or an association estimate. Possible shared provider sampling with companion LaVela reports requires consideration.	Provider accounts are not patient outcomes, patient prevalence or evidence that diet causes body-image disturbance. The convenience sample is predominantly male dietitians within VHA. Possible overlap with companion provider samples limits independent confirmation.
Pellegrini et al., 2021 [81]; Professional qualitative link	Provider accounts of weight-management behaviors, food intake, eating as coping, dietary strategies, body-composition changes and practical constraints.	Body-image concerns were explicitly identified within the empirical psychological-barriers subtheme. No patient body-image instrument was administered.	United States. Twenty-five SCI healthcare providers, including 22 women and three men, were interviewed about weight-management barriers and facilitators. Body-image concerns were identified within the empirical psychological-barriers subtheme, alongside emotional adjustment, depression and eating-related coping. Nutritional material concerned food choices, eating behaviors, weight management and practical access constraints. The body-image contribution was a brief professional thematic interpretation, without a patient instrument or detailed individual body-image assessment. The providers are not a sample of 25 patients.	The body-image contribution is a brief empirical thematic statement, not a patient scale or detailed individual account. Provider perspectives must remain distinct from patient lived experiences.
Perrier et al., 2014 [82]; Patient qualitative link, SCI subgroup	Recalled leg-muscle loss/atrophy. No objective nutritional or muscle-mass measurement.	Visual and emotional negative appraisal of changed legs, distinct from the broader loss of athletic identity.	North America. Narrative interviews included 11 adults with acquired physical disabilities, of whom nine had SCI and two had above-knee amputations. Participants were sampled from a parent cohort of 201. The full-sample age range 28–60 years and sex distribution of seven women and four men were not separately tabulated for SCI and were not transferred to that subgroup. One 52-year-old woman with paraplegia linked seeing perceived leg atrophy with intense sadness about her changed physique. This distinct subjective muscularity description and visual/emotional appraisal supported the nutrition–body-image contribution. Athletic-identity loss alone was insufficient. No objective muscle or nutritional assessment was undertaken.	Only 9 of 11 participants had SCI, with nutrition–body-image evidence anchored to an identifiable SCI account. The full-sample upper age 60 belongs to an amputee and cannot be reported as the SCI subgroup range. Perceived muscle loss is not objectively diagnosed sarcopenia, and athletic identity alone is not body-image evidence.

Report and contribution	Nutritional or eating domain	Body-image domain	Source-supported findings and denominators	Interpretive qualification
Stensman, 1989 [83]; Quantitative co-assessment	BMI calculated from actual weight and height, with individual values plotted separately for SCI. Actual body dimensions were also measured.	Situation-specific and global100-mm VAS ratings of bodily experience/satisfaction, and Body Puzzle self-estimated/actual body-dimension ratios.	Sweden. Ten adults with acquired traumatic tetraplegia were separately identifiable within 22 participants with severe mobility impairment; the remaining 12 had cerebral palsy. The SCI subgroup comprised eight men and two women aged 28–53 years. BMI was calculated from actual height and weight and plotted individually. Separate 100-mm VAS ratings assessed bodily experience and satisfaction, while the Body Puzzle compared estimated and actual body dimensions. Global body-image VAS averaged 57.2/100 in the SCI subgroup. No specific BMI–body-image association was analysed. Cerebral-palsy findings and full mixed-sample demographics were not attributed to SCI.	Only 10 of 22 disabled participants had acquired traumatic SCI. Body Puzzle results reflect body-size perception, not a clinical diagnosis. Co-assessment of BMI and body image does not establish association.
Vinoski Thomas et al., 2019 [84]; Patient qualitative link, SCI subgroup	Recalled weight gain or weight loss, perceived muscle loss and changes in body dimensions or muscularity. No objective anthropometry or nutritional assessment.	Explicit appearance evaluations, dislike/distress about changed appearance and functional-aesthetic body image.	United States. Five women with acquired SCI were separately identifiable within 15 women with visible physical disabilities. Eligible SCI ages were 27–48 years. SCI-specific accounts connected recalled weight gain, muscle loss and altered body dimensions with negative appearance evaluations. A reported loss of about 30 lb was a participant recollection, largely attributed by her to muscle loss, rather than prospective measurement. One SCI participant also reported cognitive/TBI-related disability. Eating-in-public avoidance accounts belonged to participants with cerebral palsy and were excluded from SCI extraction. Findings support qualitative links within an eligible subgroup, without prevalence or a quantitative association estimate.	Only 5 of 15 participants had SCI. All-disability pooled themes cannot automatically be attributed to SCI. Eating-in-public avoidance was described by participants with cerebral palsy and is excluded from SCI extraction. Weight and muscle changes are recalled, unmeasured descriptors.
Vázquez-Fariñas and Rodríguez-Martin, 2021 [85]; Patient qualitative link	Subjective leg thinning, perceived muscle loss/atrophy and altered muscular appearance. No objective body-composition or nutritional measurements.	Negative evaluation of appearance, nonrecognition of changed legs and avoidance of exposing them, within wider body-schema/awareness themes.	Spain. Grounded-theory interviews at the National Paraplegic Hospital included 32 adults with acquired SCI, 17 men and 15 women aged 18–65 years. The non-muscular-body theme connected perceived muscle loss and leg thinning with negative evaluations of appearance. An identifiable participant described disliking and not recognizing skinny legs and avoiding shorts. Nutritional/body-composition descriptors were subjective, without anthropometric, DXA or diagnostic sarcopenia measurements. General body-schema and body-ownership findings were not treated as nutrition-specific evidence. Single-hospital sampling and retrospective accounts constrain transferability.	Perceived atrophy is not objective sarcopenia or malnutrition. Body-schema/ownership findings without distinct muscularity and evaluative content are not nutrition-specific evidence. Single-hospital recruitment and recalled experience limit transferability.
Watson et al., 2024 [86]; Quantitative co-assessment	The EMGI Weight Management Gain composite represents perceived weight-management gains from exercise. It does not measure body weight, body fat, muscle mass, nutritional status or objective weight change. Weight-management motives alone are not nutritional outcomes.	The separate EMGI Appearance Gain subscale represents perceived appearance benefits, rather than a global clinical body-image assessment.	Australia. A cross-sectional survey included 105 physically active adults with SCI, 66 men and 39 women, with mean age 56.1 years. Separate Exercise Motives and Gains Inventory subscales assessed perceived weight-management gains and perceived appearance gains. These are composite self-reports rather than measured weight loss, body composition or nutritional status. Motives alone were not classified as nutritional outcomes. No direct association between the two gain constructs was tested. Several published pooled gain means fall below both subgroup means, and some demographic counts or percentages conflict. Consequently, the report contributes construct-level co-assessment rather than reliable magnitude, subgroup or efficacy estimates.	The study sampled physically active people, without a non-exercising comparator. Perceived gain composites are not measured weight loss, body composition or nutritional status. Pooled and subgroup means cannot be reconciled, precluding precise magnitude or subgroup claims. No direct NI–BI association or exercise efficacy can be inferred.

Report-level findings preserve eligible subgroup and respondent attribution. Recalled weight and perceived muscle change are not objective composition measurements. Co-assessment does not establish an association. Shared samples are linked separately, and no pooled independent participant count is implied. Abbreviations follow the main article.

Table S7. Methodological appraisal by report and empirical contribution

Report / contribution	Appraisal tool	Item/property judgments	Interpretive consequence
Abedi et al., 2023 [52]	COSMIN Risk of Bias checklist for PROMs (Version 3; 27 August 2024)	Content validity: Doubtful; Structural validity: Inadequate; Internal consistency: Very good; Convergent validity: Doubtful; Known-groups validity: Doubtful	COSMIN is applied only to measurement properties actually investigated. A total alpha, bilingual administration and factor analysis do not establish reliable SCI-specific nutritional/body-image domains. No study-wide COSMIN score is computed.
Astorino and Harness, 2020 [51]	JBI revised quasi-experimental tool (2024; 9 items; outcome/result-specific assessment)	J1 Y; J2 N; J3 Y; J4 U; J5 N; J6 Y; J7 U; J8 N; J9 Y	The same 25 completers are assessed at baseline, three and six months after therapy begins. There is no independent control and only one pretreatment measurement. Consistent administration supports change comparisons, but BSS/BSS-A reliability in this setting is uncertain and complete-case analysis omits four of 29 recruits. Concurrent care, natural recovery and training dose remain alternative explanations. The change analysis is appraised separately from exploratory adiposity regressions.
Astorino and Harness, 2020 — Body-fat association with body-satisfaction change [51]	JBI cohort checklist (2020; 11 items)	H1 NA; H2 Y; H3 Y; H4 U; H5 U; H6 NA; H7 U; H8 Y; H9 U; H10 N; H11 U	DXA procedures and repeatability support exposure measurement. Exposure groups are not defined and absence of a continuous satisfaction outcome at baseline is not applicable. Body-fat predictor timing, confounder selection, complete-case losses and model specification remain uncertain. The reported $r=0.33, p=0.049$ is incompatible with a conventional two-sided correlation using 25 independent observations; the source does not resolve the analysis denominator. The coefficient is retained as reported and not treated as a confirmed independent association.
Bailey et al., 2015 [58]	JBI qualitative checklist (2017; 10 items)	Q1 Y; Q2 Y; Q3 Y; Q4 Y; Q5 Y; Q6 Y; Q7 Y; Q8 Y; Q9 Y; Q10 Y	Q1–5: constructivist grounded theory, open interviewing and concurrent coding support a preliminary model. Q6–7: the team's cultural/disability positioning and discussion of preconceptions are explicit. Q8–10: participant quotations, member checking, ethics clearance and consent support the interpretation. These safeguards do not remove the selective recruitment or exclusion of previous eating disorders.
Bailey et al., 2016 [59]	JBI qualitative checklist (2017; 10 items)	Q1 Y; Q2 Y; Q3 Y; Q4 Y; Q5 Y; Q6 Y; Q7 U; Q8 Y; Q9 Y; Q10 Y	Q1–5: constructivist theory, interviews and coding are coherent. Q6: researcher gender, training and disciplinary experience are described. Q7: this report does not clearly describe active reflection on researchers' influence; a constructivist label and intercoder agreement alone are insufficient. Q8–10: quotations, participant checking and ethics/consent support findings. Reflexivity is insufficiently described in this report, although a more detailed account appears in the companion publication [58].

Report / contribution	Appraisal tool	Item/property judgments	Interpretive consequence
Bailey et al., 2018 [60]	JBI qualitative checklist (2017; 10 items)	Q1 U; Q2 Y; Q3 Y; Q4 Y; Q5 Y; Q6 U; Q7 U; Q8 Y; Q9 Y; Q10 Y	Q1: qualitative thematic analysis is described without an explicit philosophical position. Q2–5: a focus group and individual interviews appropriately explore dietary adherence experiences. Q6–7: the team's intervention involvement is evident, but its positionality and influence are not explicitly examined. Q8–10: quotations, member checking, independent coding and ethics/consent are reported. Six volunteers from 12 diet-arm participants may favor engaged respondents.
Barrett et al., 2023 [65]	JBI qualitative checklist (2017; 10 items)	Q1 U; Q2 Y; Q3 Y; Q4 Y; Q5 Y; Q6 U; Q7 U; Q8 Y; Q9 Y; Q10 Y	Q1: an explicit philosophical position is not reported. Q2–5: semi-structured interviews and the stated coding-reliability thematic approach fit the descriptive aim. Q6–7: interviewer positioning and influence on participants' sexual disclosures are insufficiently reported. Q8–10: pseudonymized quotations support themes; ethics approval and written consent are explicit. Agreement between coders is not a substitute for reflexivity.
Bassett and Martin Ginis, 2009 (men) [76]	JBI analytical cross-sectional checklist (2017; 8 items)	X1 Y; X2 Y; X3 U; X4 NA; X5 Y; X6 U; X7 Y; X8 Y	Adult/chronic SCI eligibility and the sample are described. Duplicate weight/waist/BIA measurements and questionnaire administration are standardized, although SCI-specific BIA validity remains uncertain. Body-satisfaction subscale consistency is reported; the single body-image impact item is less established. Candidate demographic and composition covariates are examined, but selecting covariates from nonsignificant bivariate results does not establish adequate confounding control. The moderation analysis concerns physical activity, while nutritional/body-image correlations are cross-sectional.
Bassett et al., 2009 (women) [77]	JBI analytical cross-sectional checklist (2017; 8 items)	X1 Y; X2 Y; X3 U; X4 NA; X5 U; X6 N; X7 U; X8 U	Eligibility, SHAPE-SCI recruitment and participant characteristics are described. Weight, supine waist and BIA protocols are specified, but SCI-specific validity of whole-body BIA and the body-image measure is insufficiently established in this small subsample. No condition threshold is being classified (X4 not applicable). Bivariate correlations do not control confounding. Eleven participants, numerous one-tailed tests and an exploratory $p<0.10$ category limit precision and require preservation of the original testing conventions.
Beckwith and Yau, 2013 [70]	JBI qualitative checklist (2017; 10 items)	Q1 U; Q2 Y; Q3 Y; Q4 Y; Q5 Y; Q6 Y; Q7 Y; Q8 Y; Q9 Y; Q10 Y	Q1: narrative analysis is specified, but philosophical premises are not explicit. Q2–5: interviews, narrative analysis and experiential interpretation match the objectives. Q6–7: the first author identifies as a woman with SCI, the second as a sex therapist; written self-reflection and participant-led interviews address insider influence. Q8/10: labeled accounts substantiate findings. Q9: James Cook University H3698 approval and written consent. Social-network recruitment limits transferability.

Report / contribution	Appraisal tool	Item/property judgments	Interpretive consequence
Buchtler et al., 2021 [68]	JBI qualitative checklist (2017; 10 items)	Q1 U; Q2 Y; Q3 Y; Q4 Y; Q5 Y; Q6 U; Q7 U; Q8 Y; Q9 Y; Q10 Y	Q1: a pilot/content-analysis approach is described, but philosophical premises remain unclear. Q2–5: the interview guide and inductive coding address body experience. Q6–7: credentials and absence of a therapeutic relationship are stated, but cultural/theoretical positioning and reflection on assumptions remain insufficient. Q8–10: identified quotations, ethics approval and consent are present; conclusions are linked to these accounts.
Chau et al., 2008 [62]	JBI qualitative checklist (2017; 10 items)	Q1 Y; Q2 Y; Q3 Y; Q4 Y; Q5 Y; Q6 Y; Q7 U; Q8 Y; Q9 Y; Q10 Y	Q1–5: material/discursive body and social-disability perspectives align with modified grounded theory and the experiential framework. Q6: authors explicitly locate their theoretical view and their physiotherapy/student research roles. Q7: memos, team review and member checking are described, but influence of researchers on accounts/interpretation is not explicitly examined. Q8/10: identified patient quotations anchor the framework. Q9: University of Toronto ethics approval. Parent-sample sex counts differ across reports.
Coggrave et al., 2012 [78]	JBI analytical cross-sectional checklist (2017; 8 items)	X1 Y; X2 Y; X3 U; X4 Y; X5 U; X6 N; X7 U; X8 U	Five-centre record-based identification, stoma indications and response coverage are reported: 149 invited, 104 respondents, 92 eligible. Dietary manipulation before surgery is recalled and custom body-image items lack demonstrated measurement properties. TSCS data are available for 71 participants rather than the full sample. Analyses are unadjusted, and the report contains inconsistent counts/percentages and recall-dependent pre/post comparisons. These are retrospective survey findings, not a prospective intervention effect or a demonstrated diet–body-image association.
Desneves et al., 2026 [69]	JBI qualitative checklist (2017; 10 items)	Q1 U; Q2 Y; Q3 Y; Q4 Y; Q5 Y; Q6 Y; Q7 Y; Q8 Y; Q9 Y; Q10 Y	Q1: implementation frameworks and thematic analysis are specified, but a qualitative philosophical position is not explicit. Q2–5: purposive interviews, verified transcripts and iterative coding fit acceptability and experience questions. Q6–7: the clinical interviewer's dual role and potential influence are acknowledged; a second coder unfamiliar with participants mitigated this. Q8–10: patient quotations, consent and ethics approval support the qualitative contribution.
Duarte et al., 2016 [75]	JBI qualitative checklist (2017; 10 items)	Q1 Y; Q2 Y; Q3 Y; Q4 Y; Q5 Y; Q6 U; Q7 U; Q8 Y; Q9 Y; Q10 Y	Q1–5: symbolic interactionism is consistent with an interpretive exploration of bodily meanings; interviews, field notes, coding and participant comparison are described. Q6–7: researchers' positions and their influence on data production are insufficiently reported. Q8–10: patient extracts anchor categories, and two institutional approvals and consent are reported.

Report / contribution	Appraisal tool	Item/property judgments	Interpretive consequence
Kathnelson et al., 2020 [61]	JBI qualitative checklist (2017; 10 items)	Q1 Y; Q2 Y; Q3 Y; Q4 Y; Q5 Y; Q6 Y; Q7 Y; Q8 Y; Q9 Y; Q10 Y	Q1–5: Giorgi's descriptive phenomenology, in-depth interviews and within-/across-case analysis fit the experiential question. Q6–7: the female interviewer's position relative to male participants and possible disclosure effects are discussed; a reflective decision journal is reported. Q8–10: direct accounts support themes; dual ethics approval and an explicit consent/withdrawal script are provided. Heterosexual male recruitment limits transferability.
Kehn and Kroll, 2009 [57]	JBI qualitative checklist (2017; 10 items)	Q1 U; Q2 Y; Q3 Y; Q4 Y; Q5 Y; Q6 U; Q7 U; Q8 Y; Q9 Y; Q10 Y	Q1: qualitative exploration/content analysis is specified without an explicit philosophical stance. Q2–5: participant-defined exercise categories, piloted telephone interviews and comparative coding fit the descriptive aim. Q6–7: experience and dual coding are reported, but researcher positioning and reflexive influence are insufficiently described. Q8–10: quotations support findings; IRB approval and consent are explicit.
Kim et al., 2013 [72]	JBI analytical cross-sectional checklist (2017; 8 items)	X1 Y; X2 Y; X3 U; X4 Y; X5 U; X6 N; X7 U; X8 U	X1–2: adult/chronic inclusion criteria, recruitment and demographics are described. X3: trained waist measurement is reported, but anatomical landmarks differ between the methods description and questionnaire instructions. X4: explicit sex-specific obesity cutoffs. X5–6: confounders for the perception comparison are not adequately specified/adjusted; obesity-risk regression addresses a different result. X7: single-item perception has no reported SCI validation. X8: categorical analysis is described, but denominator inconsistencies limit interpretation.
LaVela et al., 2021 [54]	JBI qualitative checklist (2017; 10 items)	Q1 Y; Q2 Y; Q3 Y; Q4 Y; Q5 Y; Q6 Y; Q7 U; Q8 Y; Q9 Y; Q10 Y	Q1–6: a biopsychoecological framework, multidisciplinary perspective, field-tested guide and inductive–deductive coding are described. Q7: researcher training and coder agreement are reported, but reflexive effects of researchers on accounts/interpretation are insufficiently explicit. Q8–10: participant-labeled quotations support themes and two IRB approvals are documented. Recruitment was predominantly male and region-specific.
LaVela et al., 2021 (personal meanings) [79]	JBI qualitative checklist (2017; 10 items)	Q1 Y; Q2 Y; Q3 Y; Q4 Y; Q5 Y; Q6 Y; Q7 U; Q8 Y; Q9 Y; Q10 Y	The bio-psycho-ecological framework explicitly locates the inquiry theoretically. A field-tested guide, verified transcripts and combined inductive/deductive thematic coding fit the question. The effects of researcher–participant relationships on the accounts are insufficiently described; agreement between coders is not reflexivity. Institutional approvals and participant-labeled quotations are reported. The 25-person, predominantly male regional sample shares its source interviews with the motivators/goal-setting report.

Report / contribution	Appraisal tool	Item/property judgments	Interpretive consequence
LaVela et al., 2024 [55]	JBI qualitative checklist (2017; 10 items)	Q1 Y; Q2 Y; Q3 Y; Q4 Y; Q5 Y; Q6 Y; Q7 U; Q8 Y; Q9 Y; Q10 Y	Q1–6: ontological assumptions, biopsychosocial framing, researcher disciplinary backgrounds and a detailed inductive–deductive analysis are reported. Q7: an audit trail and analytic memos are described, but active reflection on the interviewer–participant relationship and researcher influence is insufficiently explicit. Q8–10: quotations, thematic checking and institutional approval support the report.
LaVela et al., 2024b [56]	JBI qualitative checklist (2017; 10 items)	Q1 Y; Q2 Y; Q3 Y; Q4 Y; Q5 Y; Q6 Y; Q7 Y; Q8 Y; Q9 Y; Q10 Y	Q1–7: philosophical assumptions, professional/researcher differences, potential preconceptions and ongoing critical self-evaluation are explicit. A tested guide, verified transcripts and iterative coding fit the question. Q8–10: labeled provider accounts support themes and ethics approval is documented. The eating/body-dysmorphia connection represents a professional concern; patient diagnoses and medication effects were not evaluated.
LaVela et al., 2025 (poor-diet consequences) [80]	JBI qualitative checklist (2017; 10 items)	Q1 Y; Q2 Y; Q3 Y; Q4 Y; Q5 Y; Q6 Y; Q7 Y; Q8 Y; Q9 Y; Q10 Y	A descriptive thematic approach and explicit ontological/methodological assumptions fit the provider question. The disciplinary backgrounds and lack of first-hand clinical experience of most analysts are acknowledged, with continual critical self-evaluation of similarities and differences from participants. Verified transcripts, iterative coding, labeled quotations and institutional review support the analysis. Findings concern perceived consequences described by 12 providers, not observed patient diagnoses or causal dietary effects.
Li and Yau, 2006 [73]	JBI qualitative checklist (2017; 10 items)	Q1 Y; Q2 Y; Q3 Y; Q4 Y; Q5 Y; Q6 U; Q7 Y; Q8 Y; Q9 U; Q10 Y	Q1–5: an emic narrative approach, home interviews and content analysis fit the research aim. Q6: authors' cultural/theoretical positions are not sufficiently specified. Q7: personal experiences and biases were written down and discussed. Q8/10: quotations support the relevant SCI accounts. Q9: consent is reported, but formal ethical approval/exemption is not identified. SCI and poliomyelitis accounts are distinguishable for the findings considered here.
Martinsen et al., 2008 [67]	JBI qualitative checklist (2017; 10 items)	Q1 Y; Q2 Y; Q3 Y; Q4 Y; Q5 Y; Q6 Y; Q7 Y; Q8 Y; Q9 Y; Q10 Y	Q1–7: lifeworld philosophy, reflective openness and active bridling of prior knowledge are explicit; repeat interviews and meal observations support the phenomenological analysis. Q8–10: lived accounts underpin the interpretation. Written consent is documented and the local ethics committee confirmed that formal approval was not required.
Parker and Yau, 2012 [71]	JBI qualitative checklist (2017; 10 items)	Q1 U; Q2 Y; Q3 Y; Q4 Y; Q5 Y; Q6 U; Q7 Y; Q8 Y; Q9 Y; Q10 Y	Q1: descriptive interviewing/thematic analysis is specified without an explicit philosophical stance. Q2–5: in-depth interviews and within-/across-interview analysis match the experiential aim. Q6: cultural/theoretical positioning of researchers is insufficiently described. Q7: reflective diary and dialogue to address interpretive bias are reported. Q8/10: participant-labeled extracts underpin themes. Q9: James Cook University H3244 approval and consent. Four self-selected white Australian women and long recall limit transferability.

Report / contribution	Appraisal tool	Item/property judgments	Interpretive consequence
Pellegrini et al., 2021 [81]	JBI qualitative checklist (2017; 10 items)	Q1 U; Q2 Y; Q3 Y; Q4 Y; Q5 Y; Q6 U; Q7 U; Q8 Y; Q9 Y; Q10 Y	Semi-structured interviews and iterative thematic coding coherently address provider-perceived barriers and facilitators. Multiple coding and consensus are described. A philosophical stance and the analysts' beliefs or reciprocal influence on interpretation are not sufficiently explicit; disciplinary credentials alone do not establish reflexivity. Two institutional review boards are identified. Provider accounts of appearance and coping are indirect evidence of patient experience.
Perrier et al., 2014 [82]	JBI qualitative checklist (2017; 10 items)	Q1 Y; Q2 Y; Q3 Y; Q4 Y; Q5 Y; Q6 Y; Q7 Y; Q8 Y; Q9 Y; Q10 Y	Narrative inquiry, a constructionist/relativist position and dual content/structural analysis are congruent. Abductive use of theory is explicit; a critical friend challenges interpretations and requires supporting data and consideration of alternatives. Ethics approval, consent and identified accounts are documented. Nine of 11 participants had SCI. SCI-attributable muscularity and body-identity accounts remain distinct from those of amputee participants and do not quantify muscle mass.
Potgieter and Khan, 2005 [74]	JBI qualitative checklist (2017; 10 items)	Q1 U; Q2 Y; Q3 Y; Q4 Y; Q5 Y; Q6 Y; Q7 Y; Q8 Y; Q9 U; Q10 Y	Q1: qualitative/discourse rationale is given, but a philosophical position is not explicit. Q2–5: vignette-supported interviews, inclusive thematic analysis and interpretation fit the exploratory aim. Q6–7: researchers' cultural identities, prior school relationship and safeguards for voluntary recruitment are explicit. Q8/10: accounts support the themes and conclusions. Q9: parental/educational permission is described; formal ethics review or exemption is not documented.
Renwick et al., 2018 [64]	JBI qualitative checklist (2017; 10 items)	Q1 Y; Q2 Y; Q3 Y; Q4 Y; Q5 Y; Q6 Y; Q7 Y; Q8 Y; Q9 Y; Q10 Y	Q1–5: a constructivist standpoint, explicit social-interaction framework and modified grounded-theory analysis fit the question. Q6: the inquiry is located theoretically through Goffman/Garland-Thomson and constructivism. Q7: a reflexive journal examines the analyst's perspective; interpretations are discussed with supervisors. Q8/10: identified accounts support themes. Q9: Toronto approval includes secondary analyses and written consent is reported. Staring-related selection and overlap with [63] limit independence.
Rosito et al., 2002 [53]	JBI case-series checklist (2020; 10 items)	C1 Y; C2 U; C3 Y; C4 U; C5 N; C6 Y; C7 Y; C8 Y; C9 Y; C10 U	C1/3: a defined SCI/stoma database and clinical records identify the series. C2: uniform diagnostic grading for all is not fully documented. C4: consecutive participation is unclear. C5: only 27/35 identified patients responded, including survivor selection. C6–9: demographic/clinical characteristics, site and outcome domains are described. C10: mixed-domain scoring and retrospective comparisons have uncertain measurement/statistical support. One proxy and long recall further limit body-image interpretation.

Report / contribution	Appraisal tool	Item/property judgments	Interpretive consequence
Sheldon et al., 2011 [63]	JBI qualitative checklist (2017; 10 items)	Q1 Y; Q2 Y; Q3 Y; Q4 Y; Q5 Y; Q6 U; Q7 Y; Q8 Y; Q9 Y; Q10 Y	Q1–5: social-disability/psychosocial framing and modified grounded theory are consistent with the question, original interviews and analysis. Q6: researcher training is given but personal cultural/theoretical positioning is insufficiently explicit. Q7: the first author kept a reflexive journal addressing views, biases and beliefs. Q8/10: participant-labeled accounts anchor the findings. Q9: Toronto ethics approval covers secondary analysis. Age-at-injury entries are not assessment ages; overlap with [64] is retained.
Stensman, 1989 [83]	JBI analytical cross-sectional checklist (2017; 8 items)	X1 Y; X2 Y; X3 U; X4 NA; X5 U; X6 N; X7 U; X8 U	Municipal ascertainment, diagnostic groups and reference participants are described. Body-size estimation is compared with actual dimensions and BMI is reported, but anthropometric detail and measurement-property support for assisted visual ratings and the Body Puzzle are limited. No continuous-variable diagnostic threshold is being tested (X4 not applicable). Comparisons are unadjusted; mainly descriptive figures and small SCI/CP groups limit inferential interpretation. Quantitative co-assessment is appraised rather than treating unstructured narrative observations as a separate qualitative study.
Thrussell et al., 2018 [66]	JBI qualitative checklist (2017; 10 items)	Q1 Y; Q2 Y; Q3 Y; Q4 Y; Q5 Y; Q6 Y; Q7 Y; Q8 Y; Q9 U; Q10 Y	Q1–5: interpretive phenomenology, interviews and thematic analysis fit the applied experiential aim. Q6–7: clinical backgrounds, women with SCI in the team and the researchers' contribution to interpretation are acknowledged. Q8/10: age/level-labeled quotations support the findings. Q9: written consent and regulatory compliance are reported, but an approving ethics body/reference is not identified. Eligibility is restricted to separately attributable SCI accounts; sample age/CES totals differ within the source.
Vinoski Thomas et al., 2019 [84]	JBI qualitative checklist (2017; 10 items)	Q1 Y; Q2 Y; Q3 Y; Q4 Y; Q5 Y; Q6 Y; Q7 Y; Q8 Y; Q9 Y; Q10 Y	Constructivist grounded theory and feminist/disability-informed concepts fit the question. The interviewer's identity and disability position, reflexive journals, participant-guided guide changes, member checks and analytic challenge are explicitly described. Ethics approval and consent are reported. The mixed-disability sample contains five women with SCI; only their attributable accounts contribute to SCI-specific findings, and whole-sample thematic saturation cannot establish saturation within that subgroup.
Vázquez-Fariñas and Rodríguez-Martín, 2021 [85]	JBI qualitative checklist (2017; 10 items)	Q1 U; Q2 Y; Q3 Y; Q4 Y; Q5 Y; Q6 U; Q7 U; Q8 Y; Q9 Y; Q10 Y	Grounded-theory procedures, theoretical/snowball sampling and simultaneous open, axial and selective coding are congruent with the experiential aim. Participant checking and multidisciplinary analysis support the themes, but explicit philosophical premises, researcher positioning and reciprocal influence are not adequately reported. Institutional ethics approval and consent are stated. The 32-participant accounts support perceived bodily and eating changes, not measured tissue change or a causal pathway.

Report / contribution	Appraisal tool	Item/property judgments	Interpretive consequence
Watson et al., 2024 [86]	JBI analytical cross-sectional checklist (2017; 8 items)	X1 Y; X2 Y; X3 U; X4 Y; X5 U; X6 N; X7 U; X8 U	Eligibility and recruitment period are explicit; the 105-person sample is selected for recent physical activity. Guideline adherence has an explicit definition. EMGI items distinguish appearance from weight management, but SCI-specific validity and objective change are not established. Age/injury confounding is not adequately controlled. Missingness, inconsistent pooled/subgroup gain summaries and incomplete regression reporting limit numerical interpretation. Cross-sectional recalled gains cannot establish weight loss, appearance improvement or intervention efficacy.

JBI judgments are Y, Yes; N, No; U, Unclear; and NA, Not applicable. Q1–Q10 denote the qualitative checklist (2017), X1–X8 the analytical cross-sectional checklist (2017), C1–C10 the case-series checklist (2020), H1–H11 the cohort checklist (2020), and J1–J9 the revised quasi-experimental tool (2024). The latter is applied to body-satisfaction change, while the cohort checklist addresses the exploratory nutritional association within the same report. COSMIN Risk of Bias checklist version 3 (27 August 2024) ratings are property-specific, with the least favorable applicable standard determining each property rating. A favorable alpha-estimation rating does not establish unidimensionality or domain-specific validity. No summed JBI score or cross-design risk tier was calculated. Unclear ethical-review reporting does not establish unethical conduct. Abbreviations: ABT, activity-based therapy; BIA, bioelectrical impedance analysis; BMI, body mass index; BSS, Body Satisfaction Survey; BSS-A, appearance component of the Body Satisfaction Survey; COSMIN, Consensus-based Standards for the Selection of Health Measurement Instruments; DXA, dual-energy X-ray absorptiometry; IBS-QOL, Irritable Bowel Syndrome Quality of Life questionnaire; JBI, Joanna Briggs Institute; NBDS, Neurogenic Bowel Dysfunction Score; SCI, spinal cord injury; TSCS, Tennessee Self-Concept Scale. Qualitative domains are Q1 philosophical congruence, Q2 question, Q3 collection, Q4 analysis, Q5 interpretation, Q6 positioning, Q7 researcher influence, Q8 voices, Q9 ethics and Q10 grounded conclusions. Cross-sectional domains are X1 eligibility, X2 sample/setting, X3 exposure measurement, X4 condition definition, X5 confounder identification, X6 confounding control, X7 outcome measurement and X8 analysis. Case-series domains are C1 eligibility, C2 uniform assessment, C3 identification, C4 consecutive inclusion, C5 complete inclusion, C6 demographics, C7 clinical information, C8 outcomes, C9 setting and C10 analysis. Cohort domains are H1 group comparability, H2 consistent exposure assessment, H3 exposure validity/reliability, H4 confounder identification, H5 confounding control, H6 initial outcome status, H7 outcome measurement, H8 follow-up duration, H9 losses, H10 handling incomplete follow-up and H11 analysis. Revised quasi-experimental domains are J1 temporality, J2 independent control, J3 comparability, J4 concurrent care, J5 repeated pre/post measurements, J6 consistent measurement, J7 outcome reliability, J8 retention and J9 statistical analysis.

Table S8. Cohort linkage, subgroup attribution and report counts

Reports / issue	Evidence for linkage or subgroup attribution	Charting decision	Counting / interpretation
Bailey 2015 and 2016 [58,59]	The same nine participants, pseudonyms and demographic/lesion characteristics appear in companion analyses.	Positive-body-image and stigma/self-presentation findings retain their publication identities.	Two reports from one nine-person sample, not independent replication.
LaVela 2021: motivators and personal meanings [54,79]	Both reports describe 25 veterans/civilians recruited from the same Chicago sources and interviewed in late 2018, with matching sample characteristics.	Closely corresponding recruitment and interview details suggest potentially shared participants, but exact individual linkage is not established.	Two reports with potentially overlapping 25-person samples. No combined unique-patient total is inferred. The separate 25-provider study is not this patient population.
LaVela 2024 and contextual nutrition-beliefs report [55,87]	The same national sampling pool, 104 invitations, 33 veterans, 2021 interviews and matching participant identifiers support the companion link.	The eligible appearance/excess-weight findings are retained. The companion nutrition-beliefs publication supplies context but lacks an autonomous distinct body-image result.	One eligible report plus one contextual publication. The companion does not increase the 36-report corpus or establish a causal sequence.
Ontario social-adaptation project [62–64]	Chau analyzes 15 women, Sheldon 64 men and Renwick staring-related segments for 30 men from the same parent project.	The female and male contributions are distinguished; the two male analyses overlap.	Three reports. The 30-man subset is not added to the 64 men. Conflicting parent sex totals of 65/15 and 64/16 preclude reconstructing an exact unique total.

Reports / issue	Evidence for linkage or subgroup attribution	Charting decision	Counting / interpretation
SHAPE-SCI sex-specific reports [76,77]	Both use the same parent project and measurement approach; one analyzes 11 women and the other 50 men.	The samples are sex-defined and distinct within the parent project.	Two reports from different subgroups of one parent cohort. Differences between their estimates are not a tested sex interaction.
LaVela provider reports [56,80]	Both describe 12 providers from 11 Veterans Affairs facilities with similar role distributions and interview periods.	Possible shared interviews are recorded without assuming either complete identity or independence.	Two eligible professional-perspective reports. A unique provider total is not calculated while the linkage remains uncertain.
Pellegrini provider and Chicago patient studies [54,79,81]	The reports share a weight-management project and institutional setting, but sample 25 providers or 25 patients.	Provider perspectives remain distinct from the companion patient interviews.	Shared project identity does not equate professional and patient participants.
Beckwith, Parker and Li [70,71,73]	Shared authorship or research interests do not establish a participant match across the Australian and Hong Kong studies.	Each report retains its own sampling and subgroup description.	No combined unique-person total or claim of demonstrated independence is based solely on authorship.
Mixed-disability samples [73,82–84]	Eligible SCI participants comprise 10 of 22 disabled participants in Stensman, 5 of 15 in Vinoski Thomas, 9 of 11 in Perrier and 6 of 10 in Li.	Only separately attributable SCI measurements or accounts are used.	Whole-sample demographics, thematic frequencies and saturation are not automatically SCI-subgroup properties.
Thrussell 2018 [66]	The focal weight/mirror account identifies a 38-year-old participant with non-traumatic T4 AIS C SCI. The wider sample also contains CES.	The SCI-attributable account is retained; mixed SCI/CES themes are not assigned to SCI alone.	The recruited sample of 27 is not the denominator for every SCI-specific contribution. Internal age and CES-count conflicts prevent an exact eligible subgroup reconstruction.
Desneves and Abedi nested samples [52,69]	Twelve Desneves interviewees are nested within 21 clinical patients. Twelve Abedi content-validity interviewees are included among 106 patients; six professionals are separate.	Clinical, interview and professional denominators are retained for their respective findings.	Nested subsamples are not additional patients. Clinical aggregate measurements are not attributed to individual interviewees.
Bailey dietary substudy [60]	Six of 12 participants from an earlier diet intervention contributed: four individual interviews and four focus-group attendances included two people in both formats.	The eligible contribution is qualitative and uses six participants.	Eight participations do not equal eight persons. Physiological findings from the parent trial are contextual to this report.
Repeated observations and parent samples [57,67,82]	Martinsen obtained 32 interviews and 14 meal observations from 16 people. Kehn interviewed 26 members of a 592-person survey. Perrier interviewed 11 members of a 201-person parent cohort.	The number of persons is distinguished from observations and source-cohort size.	Only the report-specific eligible participants and accounts contribute to the evidence map.

The corpus contains 36 eligible journal reports, comprising 24 patient qualitative contributions, three professional qualitative contributions and nine quantitative reports. Three quantitative reports examine nutritional/body-image relationships, including one with unresolved statistical reporting; six provide co-assessment or descriptive comparisons. Categories refer to the contribution used in this review rather than all components of each study. Shared participants, repeated interviews and nested subsamples were not summed. The number of independent cohorts and unique participants was not reconstructed where overlap or subgroup boundaries remained uncertain. Abbreviations: AIS, American Spinal Injury Association Impairment Scale; CES, cauda equina syndrome; SCI, spinal cord injury; SHAPE-SCI, Study of Health and Activity in People with Spinal Cord Injury.

Table S9. Measurement limitations, reporting inconsistencies and research priorities

Report / issue	Source finding or inconsistency	Consequence for synthesis	Research priority
Astorino and Harness [51]	The source reports r = 0.33, p = 0.049, but does not specify the correlation denominator or body-fat predictor timing. The overall completer sample is 25.	The pair is incompatible with a conventional two-sided correlation test using 25 independent observations. The published result is retained as uncertain; repeated visits are not assumed to explain it.	Report the exact model, coefficient type, denominator, timing, covariates and confidence interval.
Astorino change estimates [51]	BSS means change from −0.36 to 0.44, whereas the narrative states a 0.60 increase. The source also reports r = 0.36, p = 0.03 without resolving the analysis-specific denominator.	The table-based BSS difference is 0.80; the conflicting narrative and coefficient/p pairs are not silently corrected. Satisfaction change is uncontrolled and distinct from nutritional association.	Provide reconciled endpoints and change estimates and separate prespecified from exploratory models.
Bassett women and men [76,77]	The women's pilot has n = 11 and one-tailed correlations, with an exploratory p < 0.10 category. The male report has 50 participants, waist n = 49 and differing physical-activity denominators.	The reported testing convention is preserved. Weak or nonsignificant estimates without confidence intervals do not establish absence of a relationship or a difference between sexes.	Use adequately sized sex/gender-inclusive samples, common protocols, effect intervals and explicit interaction analyses.
Coggrave [78]	Eligible respondents number 92, while TSCS data are reported for 71. The body-image passage reports 83 (70%), which is not internally consistent with the eligible denominator.	Custom body-image items, physical self-concept and dietary history are mapped separately. The inconsistent count/percentage is not presented as a verified prevalence.	Report item-specific denominators, missingness, validated bodily-evaluation measures and prospective dietary data.
Watson [86]	The reported pooled weight-management gain mean differs from both subgroup means in a way that cannot be reconciled from the article. Other appearance summaries, regression statistics and missingness also vary.	The study contributes perceived motives and gains, not verified weight loss or appearance improvement. Precise pooled/subgroup gain magnitudes are not relied upon.	Reconcile scoring and denominators, publish complete model output and collect actual nutritional and body-image outcomes prospectively.
Kim [72]	Enrollment is 371; the explicit abdominal-obesity subgroup is 84. Normal-waist totals, sex cells, some injury counts and waist landmarks conflict.	The 84-person subgroup is used for its body-perception and dieting percentages. The stated 29.2% obesity prevalence is not recalculated using enrollment or an inferred complete-case total.	Use one reproducible waist protocol and report denominators, missingness and SCI-specific validity for every analysis.
Abedi [52]	The IBS-QOL total alpha is 0.9684. Structural testing uses 106 participants for 33 retained items, with CFI = 0.6149 and RMSEA = 0.1462 for the alternative model.	High total alpha does not establish unidimensionality, body-image domain reliability or a nutrition/body-image relationship. The structural-validity methods remain limited under COSMIN.	Evaluate content, dimensionality and score-specific reliability before relating separate nutritional and body-image domains.
Abedi subgroup and comparator data [52]	Twelve patients are nested in the sample of 106. Language percentages and some subgroup descriptors conflict; NBDS correlations concern bowel dysfunction and satisfaction.	Counts are distinguished from inconsistent percentages. Total bowel/QOL correlations are not relabeled as associations between nutritional and body-image domains.	Report consistent subgroup descriptors and distinguish score validation from the substantive relationships tested.
Rosito [53]	The body-image comparison is printed as t = 0.369, p < 0.715 and described as non-significant. Dietary history and pre-stoma change are recalled; weight-loss means concern eight pre-stoma and two post-stoma respondents.	The source's non-significant interpretation is retained with its statistical-printing inconsistency acknowledged. Neither whole-sample weight change nor a causal stoma effect is inferred.	Use prospective measurements, explicit domain denominators and consistent statistical reporting.
Desneves [69]	Clinical n = 21, patient interviews n = 12, trained clinicians n = 6 and focus-group clinicians n = 5 refer to different units. Age IQR is 45–64 in the table and 54–64 in prose.	Clinical summaries are contextual to the interviewed subgroup. Table-based age dispersion is preferred if reported, and acceptability is not treated as efficacy.	Link qualitative and quantitative observations by participant and time, with consistent subgroup reporting.

Report / issue	Source finding or inconsistency	Consequence for synthesis	Research priority
Stensman [83]	Ten participants have acquired SCI and 12 have cerebral palsy; a separate 43-person reference group is described. BMI, body-size perception and satisfaction are co-assessed.	The pooled disabled sample is not an SCI sample. BMI or perceived size alone does not establish dissatisfaction, nutritional pathology or a statistical relationship.	Report group-specific results with supported measurement methods and uncertainty.
Mixed qualitative samples [66,73,82,84]	Pooled themes may combine SCI with cerebral palsy, amputation, poliomyelitis or CES. The oldest participant in Perrier's full sample is an amputee.	Only attributable SCI accounts enter the synthesis. Pooled age ranges, eating-avoidance accounts or thematic frequencies are not transferred to the SCI subgroup.	Provide participant-level diagnostic attribution and subgroup-specific characteristics while preserving confidentiality.
Adolescent evidence [74,75]	Potgieter includes seven participants aged 15–20. Duarte includes only two adolescents among 12 participants and gives inconsistent lower bounds for the adolescent age band.	Neither study provides a representative adolescent estimate. Age at injury and current assessment age are distinguished, and the whole Duarte sample is not described as adolescent.	Recruit developmentally defined groups and report assessment age, injury timing, sex/gender and nutritional/body-image measures separately.
Perceived morphology across qualitative reports	Participants describe weight gain/loss, thinner limbs, muscularity or abdominal change alongside attractiveness, acceptance or self-presentation.	These descriptions support experiential findings rather than measured adiposity, sarcopenia, malnutrition or tissue-specific mechanisms.	Combine appropriate clinical measurements with interviews and distinct bodily-evaluation outcomes.
Temporality and intervention claims	Longitudinal nutritional/body-image analysis is sparse. Linked interviews, cross-sectional correlations and recalled gains do not establish temporal order.	The map does not show that dissatisfaction causes restriction or that altering nutrition or composition improves body image.	Prespecify temporal hypotheses, repeat both domains, justify confounder sets and evaluate candidate interventions prospectively.
Appraisal and evidence coverage	Qualitative reporting varies in reflexivity and ethical-review detail; quantitative work has confounding, attrition and measurement limitations. Psychological and nursing coverage increased, while some interface metadata remain incomplete.	The appraisal informs the scope of interpretations rather than an exclusion score. The map remains bounded by English-report eligibility, source coverage and documented search metadata.	Improve reflexive reporting, measurement validity and missing-data methods, and extend recruitment and search coverage to underrepresented populations and settings.

Published values are retained as reported when a discrepancy cannot be resolved from the source. Alternative denominators, corrected coefficients or unreported subgroup results are not inferred. Differences between measurement, perception and recalled change inform the scope of each interpretation. Abbreviations: BIA, bioelectrical impedance analysis; BMI, body mass index; BSS, Body Satisfaction Survey; BSS-A, appearance component of the Body Satisfaction Survey; CFI, comparative fit index; COSMIN, Consensus-based Standards for the Selection of Health Measurement Instruments; EMGI, Exercise Motives and Gains Inventory; IBS-QOL, Irritable Bowel Syndrome Quality of Life questionnaire; IQR, interquartile range; NBDS, Neurogenic Bowel Dysfunction Score; QOL, quality of life; RMSEA, root mean square error of approximation; SCI, spinal cord injury; TSCS, Tennessee Self-Concept Scale.

Table S10. Conceptual applications of the evidence map to coordinated rehabilitation assessment

Illustrative scenario	Assessment topics	Conceptual coordination and distinct outcomes	Evidence basis and limits
Abdominal appearance concerns with mirror or clothing avoidance	Weight trajectory, eating adequacy and possible bowel/postural contributors could be considered alongside the meaning of bodily change and effects on dressing or intimacy.	Patient priorities could guide coordinated dietetic, rehabilitation, psychological and occupational assessment. Nutritional findings, distress and chosen participation goals would remain separate outcomes.	SCI accounts identify the concern [62,65,66,70,71]. The combined pathway has not been evaluated.
Meals skipped before an outing or therapy	An assessment could distinguish actual intake, bowel fears, assistance availability and any appearance-related motivation.	Dietetic, nursing and occupational input could explore a feasible meal/bowel routine, with psychological assessment when relevant. Adequacy, symptoms and attendance would be monitored separately.	Eating, bowel and presentation findings provide distinct contextual contributions [53,67,78]; bowel guidance supplies clinical context [107]. A causal body-image motive is not assumed.
Perceived loss of muscularity changes attractiveness	Perceived tissue change, personal muscularity ideals and valued bodily capacities could be explored without equating weakness with muscle loss.	Any clinical assessment could use appropriate nutritional/composition measures and patient-chosen rehabilitation goals. Appearance distress and meaningful activity would remain distinct from measured tissue change.	Qualitative SCI accounts support the topic [61,63,68,82]. Functionality literature is indirect for SCI [117].
Severe dietary restriction accompanies a wish to look thinner	Intake, weight trajectory, medical consequences and the person's reasons for restriction could be assessed without assigning an eating-disorder diagnosis from a theme.	A coordinated dietetic and psychological assessment could consider nutritional needs, distress and participation goals. Diagnostic decisions would require their own assessment.	Patient weight-management accounts and professional concerns are distinct evidence [54,56,80]. Mental-health guidance provides context [116]. The map does not establish prevalence or causal sequence.
Public assisted eating feels undignified	Food choice and intake could be considered with embarrassment, control, preferred pace and assistance.	Occupational, nursing and dietetic input could consider patient-selected adaptations. Adequacy, perceived control and chosen social-meal participation would be evaluated separately.	Lifeworld interviews and meal observations support the experiential topic [67]. Food-access literature adds context [98,99]. Adaptations require individual evaluation.
Weight-loss goals coexist with wound-related nutritional needs	Current nutritional requirements, intake, weight goals and distress about shape could be considered together.	Wound and dietetic assessment could be coordinated with relevant psychological support. Adequacy, clinical outcomes and distress would remain separate endpoints.	External pressure-injury nutrition research supplies clinical context [96]. This is an illustrative assessment problem, not an intervention result of the included corpus.
An adolescent describes thinness as unattractive	Developmental nutritional needs, recalled weight change, peer experiences and preferences for family involvement could be explored using age-appropriate methods.	The young person's priorities could inform coordinated nutrition and rehabilitation assessment. Growth/nutrition, body-related distress and participation would remain distinct outcomes.	Small adolescent/young-adult studies identify this topic [74,75]. Adult intervention findings are not assumed transferable, and the proposed coordination is untested.

These scenarios are untested applications of the evidence map, not additional participant cases or interventions evaluated by the review. They identify possible assessment topics rather than validated clinical pathways or recommendations for routine implementation. Their acceptability, feasibility and effects require evaluation. Patient priorities and applicable clinical guidance would inform any assessment, with nutritional, psychological and participation outcomes considered separately. Abbreviation: SCI, spinal cord injury.
